# Enhancement of Female Rat Fertility via Ethanolic Extract from *Nigella sativa* L. (Black Cumin) Seeds Assessed via HPLC-ESI-MS/MS and Molecular Docking

**DOI:** 10.3390/molecules29030735

**Published:** 2024-02-05

**Authors:** Ahmed M. Nagy, Mohamed F. Abdelhameed, Asmaa S. Abd Elkarim, Tushar C. Sarker, Ahmed M. Abd-ElGawad, Abdelsamed I. Elshamy, Abdelmohsen M. Hammam

**Affiliations:** 1 Department of Animal Reproduction & AI, Veterinary Research Institute, National Research Center, Cairo 12622, Egypt; am.hammam@nrc.sci.eg; 2 Department of Pharmacology, National Research Center, Cairo 12622, Egypt; fayed.nrc@gmail.com; 3 Chemistry of Tanning Materials and Leather Technology Department, National Research Center, Cairo 12622, Egypt; as.abdel-maksoud@nrc.sci.eg; 4 Texas A&M AgriLife Research Center, Overton, TX 75684, USA; tushar.sarker@ag.tamu.edu; 5 Plant Production Department, College of Food & Agriculture Sciences, King Saud University, P.O. Box 2460, Riyadh 11451, Saudi Arabia; aibrahim2@ksu.edu.sa; 6 Department of Natural Compounds Chemistry, National Research Center, Cairo 12622, Egypt

**Keywords:** *Nigella sativa*, HPLC-ESI-MS/MS, LH, FSH, E2 hormones, molecular docking

## Abstract

The characteristic chemical composition of *Nigella* seeds is directly linked to their beneficial properties. This study aimed to investigate the phytochemical composition of *Nigella sativa* seeds using a 100% ethanolic extract using HPLC-ESI-MS/MS. Additionally, it explored the potential biological effects of the extract on female rat reproduction. Follicle Stimulating Hormone (FSH), Luteinizing Hormone (LH), Estrogen (E2), and Progesterone (P4) hormone levels were also assessed, along with the morphological and histological effects of the extract on ovarian, oviductal, and uterine tissues. Molecular docking was performed to understand the extract’s activity and its role in regulating female reproduction by assessing its binding affinity to hormonal receptors. Twenty metabolites, including alkaloids, saponins, terpenes, flavonoids, phenolic acids, and fatty acids, were found in the ethanolic extract of *N. sativa* seeds through the HPLC-ESI-MS/MS study. The *N. sativa* seed extract exhibited strong estrogenic and LH-like activities (*p* < 0.05) with weak FSH-like activity. Furthermore, it increased the serum levels of LH (*p* < 0.05), P4 hormones (*p* < 0.001), and E2 (*p* < 0.0001). Molecular docking results displayed a strong interaction with Erβ, LH, GnRH, and P4 receptors, respectively. Based on these findings, *N. sativa* seeds demonstrated hormone-like activities, suggesting their potential as a treatment for improving female fertility.

## 1. Introduction

Female infertility is a widespread condition that affects many women worldwide. It refers to the inability to conceive or sustain a pregnancy after trying for at least one year. Various factors can cause infertility, including hormonal imbalances, structural abnormalities, and lifestyle choices. The emotional impact of infertility can be devastating, affecting a woman’s self-esteem, relationships, and overall quality of life [1]. Hormones, such as Follicle Stimulating Hormone (FSH), Luteinizing Hormone (LH), Estrogen (E2), and Progesterone (P4), play a vital role in female reproduction, working together like a symphony orchestra. FSH promotes the growth and development of immature oocytes, stimulates follicle maturation, and supports follicular growth [2,3]. LH triggers ovulation and the formation of corpora lutea, leading to Progesterone production [4]. P4 is crucial for maintaining pregnancy, releasing mature oocytes, and facilitating implantation [5,6,7]. E2 is essential for the growth and activation of the female reproductive system, as well as the development of secondary sex characteristics [8]. Disruptions in the production or action of these hormones can interrupt the reproductive process and potentially lead to infertility. In recent times, medicinal plants have gained significance in clinical trials and in the treatment of various diseases due to their economic price and high safety compared to chemical therapy [9]. 

*Nigella sativa* L., commonly known as black cumin, is a traditional medicinal plant belonging to the Ranunculaceae family [10]. It has various names, such as black seed, Al-habbah Al-Sawda, and Habbet el-Baraka in Arabic and Mediterranean Sea countries [11]. In India and Pakistan, it is called Kalonji, while in America, it is known as black cumin [12,13]. *N. sativa* seeds are widely used in cooking due to their nutritional value, containing proteins, carbohydrates, vitamins, minerals, and fats [14]. The seeds of *N. sativa* contain several chemical components, such as flavonoids, saponin, phenolic acids, alkaloids, essential and fixed oils, and proteins [14,15,16]. Its pharmacological and therapeutic benefits can be attributed to the high concentration of beneficial compounds, including thymoquinone (TQ), thymohydroquinone, thymol, carvacrol, nigellidine, nigellicine, and α-hederin [17]. These bioactive ingredients contribute to its remarkable biological properties, positioning *N. sativa* as a promising natural remedy for disease prevention and management [18].

For over 2000 years, *N. sativa* seeds have been widely used in Middle Eastern and Arabic traditional medicines to treat various ailments, such as skin conditions, stomach issues, headaches, diabetes, rheumatism, fever, cough, hypertension, and influenza [19,20,21]. Numerous studies have focused on the use of *N. sativa* for improving immunity, cardiovascular health, and digestive function, as well as treating complications, such as obesity, hypertension, and diabetes [9,22,23,24]. Moreover, research studies have demonstrated the antioxidant, antibacterial, antiparasitic, anti-inflammatory, and anticarcinogenic effects of *N. sativa* seeds [12,25,26]. Previous studies have shown that *N. sativa* seeds have a positive impact on the male reproductive system and hormonal balance [10,27]. However, there is limited understanding of how *N. sativa* affects the female reproductive system and hormonal balance. Therefore, the objectives of this study were to (i) investigate the effects of an ethanolic extract of *N. sativa* seeds on hormonal levels and the structural makeup and histological appearance of the genital system in immature female rats; (ii) evaluate the phytochemical profile using LC-MS-MS analysis to better understand the action mechanism; and (iii) conduct in silico molecular docking studies to investigate the function of the identified compounds.

## 2. Results

### 2.1. Qualitative Characterization of Phytoconstituents in N. sativa seed Ethanol Fractional Extract through HPLC/ESI-MS/MS Analysis

Twenty metabolites were identified via HPLC-ESI-MS/MS analysis of the *N. sativa* seed ethanol extract. The identification was performed depending on the mass data in a negative mode and a comparison with previously documented data [28,29]. All of the identified compounds, along with the R_t_, observed mass ion peak, and major mass fragmentation ions of each, were inserted in Table 1. LC-MS spectral interpretation allowed for the identification of four alkaloids, magnoflorine, nigellidine, dihydronigellidine, and nigelanoid; four saponins, hederagenin, hederageninpentoside, tauroside E, and hederagenin methyl ester; three monoterpenes, thymoquinolglucoside, thymoquinone, and thymoquinolglucuronic acid; one flavonoid, kaempferol; four phenolic acids, vanillic acid, methoxy cinnamic acid, caffeic acid 3-*O*-glucuronide, and (*E*)-*p*-coumaric acid; and three fatty acids, marked by their MS/MS and in negative ionization mode.

#### 2.1.1. Alkaloids

Compound **3** showed a molecular ion peak at *m*/*z* 340.08, and it was tentatively identified as magnoflorine (Figure S1). The component showed deprotonated [M-H]^−^ at *m*/*z* 295.17, resulting in a characteristic fragment at *m*/*z* 277.28 [M-H-H_2_O]^−^, suggested as nigellidine (5) (Figure S2). Compound **10**, detected at R_t_ 27.012 min, showed a precursor ion [M-H]^−^ at *m*/*z* 293.07 and product ions at 265.05 [M-H-CO]^−^, 237.05 [M-H-2CO]^−^, and 222.09 [M-H-CO-CH_3_]^−^, representing dihydronigellidine (Figure S3) [28]. Compound **18**, with the precursor ion [M-H]^−^ at *m*/*z* 312.11 and ions at *m*/*z* 293.86 [M-H-H_2_O]^−^, 275.86 [M-H-H_2_O]^−^, 183.04, and 119.08, was proposed as nigelanoid (Figure S4) [31].

#### 2.1.2. Saponins

Compound **6** was tentatively identified as a hederagenin aglycone with a precursor ion at *m*/*z* 471.73 and a fragment ion at *m*/*z* 393.02 after the loss of [M-H-C_2_H_6_O_3_]^−^. A precursor ion at *m*/*z* 749.20 gave fragment ions at *m*/*z* 603.29 [M-H-Rha (146)]^−^ and 471.36 [M-H-Rha-Pent (308)]^−^ due to loss of rhamnose and pentose sugar, which were suggested as hederageninpentoside (**7**) and tauroside E (**8**) (Figure S5), respectively [30,31,32]. Compound 9, at R_t_ 18.390 min, was detected as hederagenin methyl ester via the deprotonation [M-H]^−^ at *m*/*z* 485.21 alongside the product ions at *m*/*z* 467.56 [M-H-H_2_O]^−^ and 439.83[M-H-H_2_O-CO]^−^ (Figure S6) [28,32].

#### 2.1.3. Monoterpenes

Three thymoquinone derivatives were proposed for compounds **4, 12,** and **16** at *m*/*z* 325.05, 163.98, and 339.16 at different R_t_ at 8.423, 31.689, and 33.622, respectively [29,30]. For compound 4, the ions were observed in the MS/MS spectra with fragment ions at *m*/*z* 310.07 [M-H-CH_3_]^−^, 282.09 [M-H-CH_3_-CH-CH_3_]^−^, and 163.00 [M-H-glucose (162)]^−^, corresponding to thymoquinolglucoside (Figure S7) [28,32]. Meanwhile, compound **12**, with the product ions at *m*/*z* 117.92, was tentatively identified as thymoquinone (Figure S8). Also, compound **16** at *m*/*z* 339.16 and its production at 163.12 [M-H-glucuronic acid (176)]^−^ was annotated as thymoquinolglucuronic acid (Figure S9) [32].

#### 2.1.4. Phenolic Acids

The -ve ion mode HPLC-ESI-MS/MS analysis revealed the presence of four phenolic-like structures (**2, 11, 13**, and **14**), including vanillic acid (**2**) and another three phenolic acids. Depending on a molecular ion peak at *m*/*z* 163.07 and a characteristic product ion at m/z 119.00 [M-H-CO_2_]^−^, compound 14 was annotated as €-*p*-coumaric acid. While the molecular ion peak at *m*/*z* 117.87, along with the fragment ion 159.90 [M-H-H_2_O]^−^, allowed for the assignment of compound 11 as methoxy cinnamic acid, based upon the molecular ion peak at *m*/*z* 355.3 and the product ions 179.14 [M-H-glucuronic acid (176)]^−^ and 163.15 [M-H-glucuronic acid-H_2_O]^−^, it was suggested that compound 13 is caffeic acid 3-*O*-glucuronide (Figure S10). Phenolics commonly form a pseudo-molecular ion [M-H]^−^ corresponding to a deprotonated molecule and a characteristic fragment ion [M-H-44]^−^ related to CO_2_ loss from the carboxylic acid group [30,31].

#### 2.1.5. Fatty Acids

Based on the negative ionization MS in the second half of the chromatographic run, numerous fatty acids were assigned as significant peaks. Octadecadienoic acid (**17**) (Figure S11), octadecenoic acid (**19**) (Figure S12), and palmitic acid (**20**) were straightforward to interpret and had exact masses of 279.24, 281.22, and 255.29, respectively [25,26]. These were the least polar metabolites of all chromatograms.

### 2.2. Biological Results

#### 2.2.1. Evaluation of FSH-like Activity

To assess the FSH-like activity, we conducted various evaluations, including examining the gross morphological features of the genitals, measuring the weight of the ovaries and genitals, determining serum FSH levels, and conducting a histological examination of the genitals.

##### Ovarian and Uterine Weights

**Figure 1:**
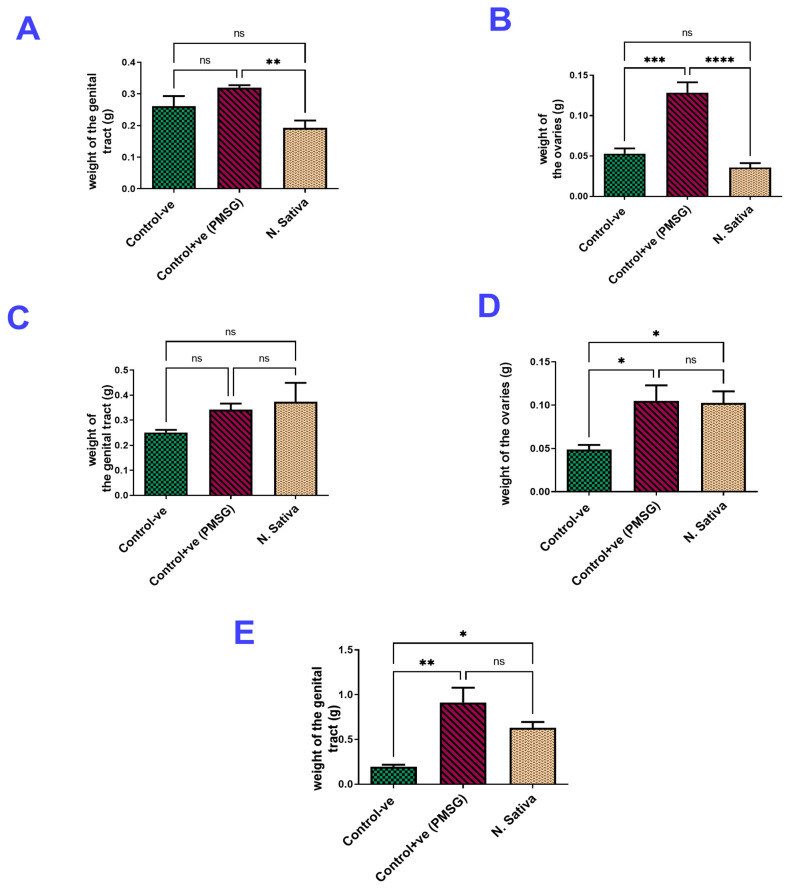
Weight of the genitalia and the ovaries of rats showing (**A**,**B**) the FSH-like activity of *N. sativa* seeds. (**C**,**D**) The LH-like activity of *N. sativa* seeds. (**E**) Weight of the uterine tissue/g, showing the Estrogen-like activity of *N. sativa* seeds (mean ± SEM, ^ns^ non-significant, * *p* ≤ 0.05, ** *p* ≤ 0.01, *** *p* ≤ 0.001, **** *p* ≤ 0.0001).

The results of the genitalia did not show any significant differences between the group treated with *N. sativa* and the negative control group. Both groups had similar weights for the genitalia and ovaries. However, the group treated with PMSG (20 IU) showed a highly significant increase in the weight of the genitalia (*p* < 0.05) and the ovarian weight (*p* < 0.001) compared to both the control and *N. sativa* groups (Figure 1A,B).

##### Morphological Examination of Genitalia

**Figure 2:**
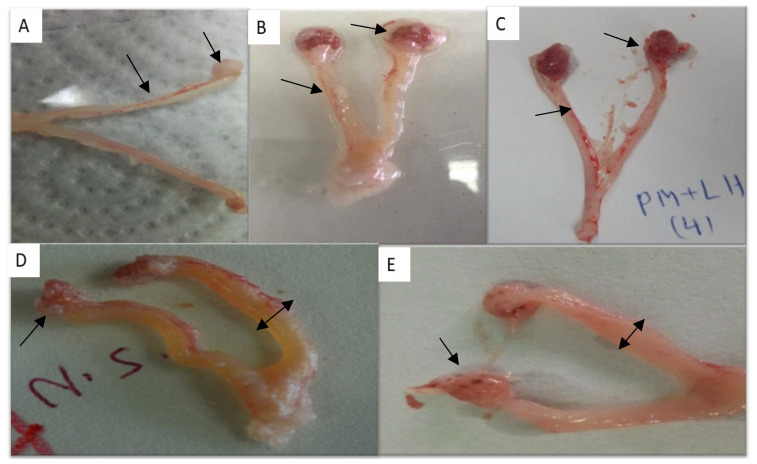
Female immature rats. (**A**) Control -ve group treated with saline; the uterus is elongated and thin, and the ovaries are small and contain small numbers of follicles (the arrows). (**B**) Control FSH +ve group treated with 20 IU of PMSG. The uterine body and horns are wide in diameter and filled with large quantities of fluids, and the ovaries are large and contain large numbers of large follicles (the arrows). (**C**) Control LH +ve group treated with 20 IU of PMSG followed by 20 IU of hCG 3 days later. The uteri were short and contained a large amount of secretions. The ovaries contained numerous corpora lutea (the arrows). (**D**,**E**) The uterine body and horns in the *N. sativa*-treated group were short, large in diameter, and filled with uterine secretions. The ovaries contained a large number of corpora lutea (CLs) (the arrows).

In the negative control group of immature female rats treated with saline, the uterus appeared elongated and thin, and it contained a small volume of fluids (Figure 2A). The ovaries were small in size and had a low number of follicles. In the group treated with PMSG (20 IU), the uterine body and horns were wide in diameter and filled with a large volume of fluids (Figure 2B). The ovaries were large and contained a large number of large follicles. Similarly, immature female rats treated with the ethanolic extract of *N. sativa* exhibited a significantly enlarged uterus filled with a substantial amount of uterine secretions. The ovaries in this group contained large follicles (Figure 2D,E).

##### Histological Examination

**Figure 3:**
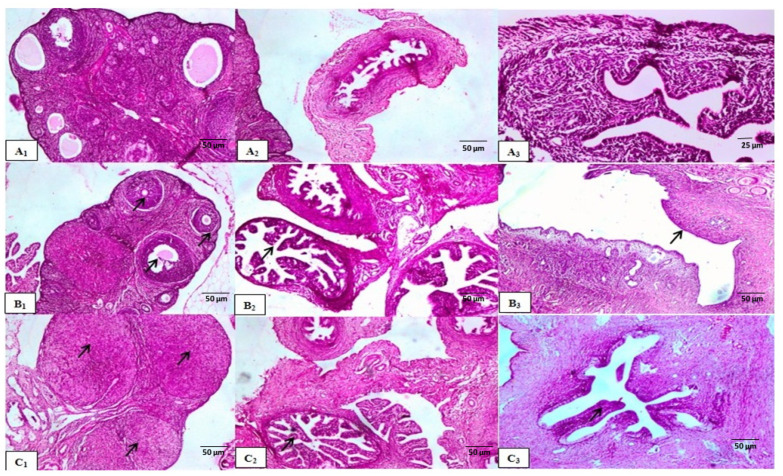
Cross-sections of the ovarian, oviductal, and uterine tissues (magnification 40×) of (**A1**–**A3**) control –ve group. (**B1**–**B3**) Control +ve FSH-like activity (PMSG) showing different stages of follicular maturation and large number of mature follicles (arrow). (**C1**–**C3**) Control +ve LH-like activity (PMSG + LH) showing strong luteal activity in the ovaries (arrow), characterized by the presence of multiple adjacent well-developed CLs and potential endometrial activity (arrow), including an increase in endometrial epithelium height.

**Figure 4:**
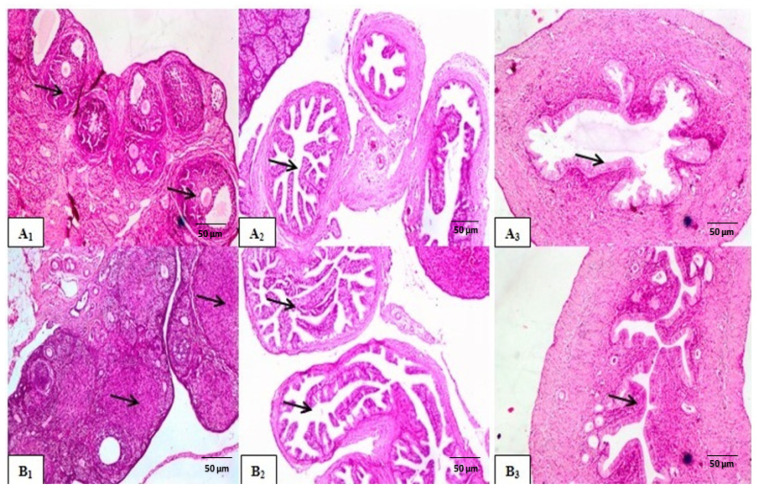
Cross-sections of the ovarian, oviductal, and uterine tissues (magnification 40× and 100×) revealed (**A1**–**A3**) FSH-like activity of *N. sativa* characterized by the presence of multiple active follicles at different developmental stages (arrow). (**B1**–**B3**) LH-like activity of *N. sativa* showing multiple CLs and progestational proliferation, characterized by columnar lining epithelium and folding of endometrial villi (arrow).

The histological micrographs of the control group displayed a normal follicular cycle in the ovaries, with follicles at various developmental stages, including growing, mature, and atretic follicles associated with corpus albicans (Figure 3(A1)). In contrast, the reference group showed different stages of follicular maturation in the ovarian cortex with large number of mature follicles (Figure 3(B1)). However, the group treated with *N. sativa* exhibited weak FSH-like activity compared to the control group, as evidenced by the presence of multiple active follicles at different developmental stages (Figure 4(A1)). Histological examination of the uterine tissues in the reference group revealed progestational proliferation, characterized by an increasing in the height of epithelial lining and folding of endometrial villi, compared to the control group (Figure 3(B2,B3)). Similarly, the *N. sativa*-treated group exhibited moderate luteal activities, including an increase in endometrium thickness, proliferation of luminal epithelial cells, dilation of uterine glands, and congestion of blood capillaries (Figure 4(A3,B3)). In summary, histological examination showed that the reference and *N. sativa*-treated groups exhibited strong luteal activity in the ovaries, characterized by the presence of well-developed corpora lutea. The uteri of both the reference and *N. sativa* groups showed potential endometrial activity, including an enlargement of the uterus, glandular proliferation, an increase in endometrial epithelium height, and corrugation and folding of endometrial villi, resembling progestational proliferation.

#### 2.2.2. Evaluation of LH-like Activity

##### Ovarian and Uterine Weights 

There were no significant changes observed in the weight of the genitals between the negative control group, the positive control group, and the group treated with *N. sativa* (Figure 1C). However, the weight of the ovaries was significantly increased in both the standard LH group and the *N. sativa*-treated group (*p* < 0.05) compared to the negative control group (Figure 1D).

##### Morphological Examination of Genitalia

In the negative control group, immature female rats displayed elongated uterine horns with small diameters containing small quantities of fluids, and the ovaries contained a small number of corpora lutea (Figure 2A). The positive control group, which received PMSG and hCG, (PMSG 20 IU/mL and hCG 5 IU/rat), exhibited elongated uterine horns with small diameters containing small quantities of fluids, and the ovaries were large, with a small number of corpora lutea (Figure 2C). In immature female rats treated with *N. sativa*, the uterine horns were short, large in diameter, and filled with uterine secretions, and the ovaries were large and contained a large number of corpora lutea (Figure 2D,E).

##### Histological Examination

Histological examination revealed that the reference group, which received PMSG followed by hCG, exhibited strong luteal activity in the ovaries, characterized by the presence of multiple well-developed corpora lutea adjacent to each other (Figure 3(C1)). Similarly, the *N. sativa*-treated group also showed strong luteal activity, indicating an active follicular cycle (Figure 4(B1)). In terms of uterine activity, both the reference group (treated with Folone 0.02 mg/kg) and the *N. sativa* group demonstrated potential endometrial activity, including an enlargement of the uterus, glandular proliferation, an increase in endometrial epithelium height, and corrugation and folding of endometrial villi, resembling progestational proliferation (Figure 3(C3) and Figure 4(B3)).

#### 2.2.3. Evaluation of Estrogen-like Activity

##### Ovarian and Uterine Weights

The ovariectomized immature female rats treated with *N. sativa* ethanolic extract and standard E2 showed a significant increase in the uterine weight (*p* < 0.05, *p* < 0.01, respectively) compared to the control ovariectomized rats, as shown in the data above in Figure 1E.

##### Morphological Examination of Genitalia

**Figure 5:**
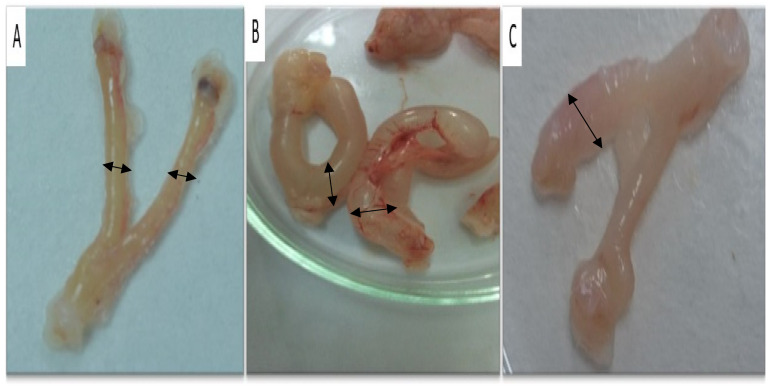
Ovariectomized female rats. (**A**) Control (–ve) group treated with saline. The uterus is thin and elongated. (**B**) Control (+ve) group treated with Folone^®^ (E2). The uterus is short, thin, and filled with large amounts of fluid, showing estrogenic activity. (**C**) *N. sativa*-treated group. The uterus is short and filled with medium amounts of secretions, showing estrogenic activity. The arrows showing the difference of uterine thickness between the groups.

In the morphological examination of genitalia, ovariectomized female rats treated with saline (negative control group) displayed an elongated uterus filled with a moderate amount of fluid (Figure 5A). In contrast, ovariectomized female rats treated with Folone (positive control group) had a short uterus with a thin membrane filled with large amounts of fluid, indicating estrogenic activity (Figure 5B). Ovariectomized female rats treated with the ethanolic extract of *N. sativa* exhibited a short uterus filled with a moderate amount of secretions, indicating moderate estrogenic attributes of the plant extract (Figure 5C).

##### Histological Examination

**Figure 6:**
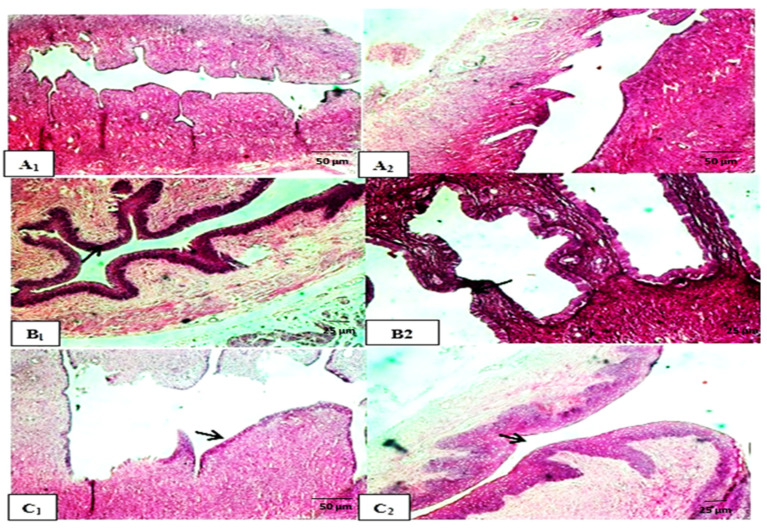
Cross-sections of the uterine tissues (magnification 40× and 100×) revealed the estrogenic activity of (**A1**,**A2**) control ovariectomized rats. (**B1**,**B2**) E2-like activity of Folone-injected group. showing strong uterine activity in the reference group, characterized by active endometrial hyperplasia and dilation of uterine glands (arrow). (**C1**,**C2**) E2-like activity of *N. sativa*-treated group showing good estrogenic activity, as evidenced by the presence of active endometrial hyperplasia (arrow).

Histological micrographs of ovariectomized rats showed strong uterine activity in the reference group, characterized by active endometrial hyperplasia and dilation of uterine glands compared to the control group (Figure 6(A1,A2,B1,B2)). Similarly, the *N. sativa*-treated group exhibited good estrogenic activity, as evidenced by the presence of active endometrial hyperplasia and the dilation of uterine glands with the infiltration of inflammatory cells (Figure 6(C1,C2)).

##### Determination of Serum Hormones

**Figure 7:**
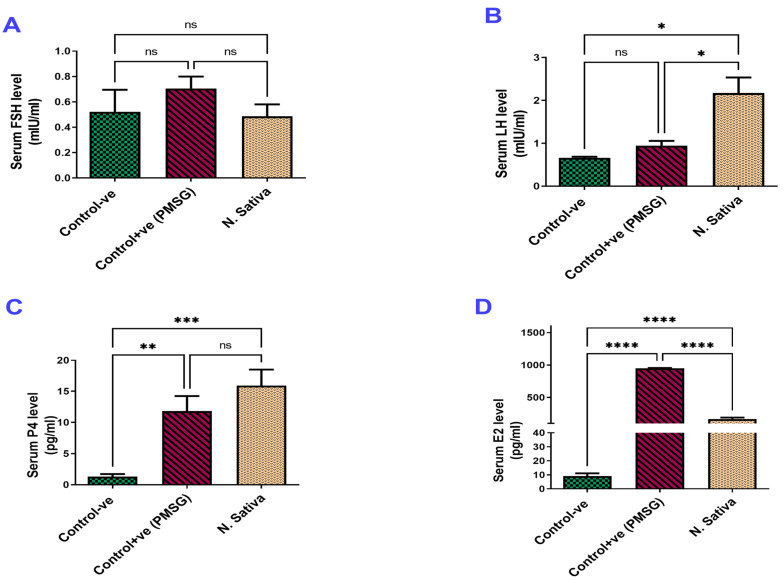
Concentrations of the gonadotropins and steroidal hormones in the serum of rats. (**A**) FSH serum concentration (mIU/mL). (**B**) LH concentration (mIU/mL). (**C**) Estrogen (E2) concentration (pg/mL). (**D**) Progesterone (P4) concentration (pg/mL), (mean ± SEM, ^ns^ non-significant, * *p* ≤ 0.05, ** *p* ≤ 0.01,*** *p* ≤ 0.001, **** *p* ≤ 0.0001).

The results revealed that the FSH levels of the female immature rats treated with *N. sativa* extract did not change significantly with the control group (Figure 7A). Meanwhile, the serum LH level was significantly higher (*p* < 0.05) in the *N. sativa*-treated group compared to the control and standard groups (Figure 7B). In the same way, the serum P4 levels were significantly higher (*p* < 0.001) in the *N. sativa*-treated group and the standard LH group compared with the control group (Figure 7C). Moreover, the serum E2 levels were significantly higher (*p* < 0.0001) in the *N. sativa*-treated group and the standard E2 group compared to the control group, as shown in Figure 7D.

##### Molecular Docking 

**Figure 8:**
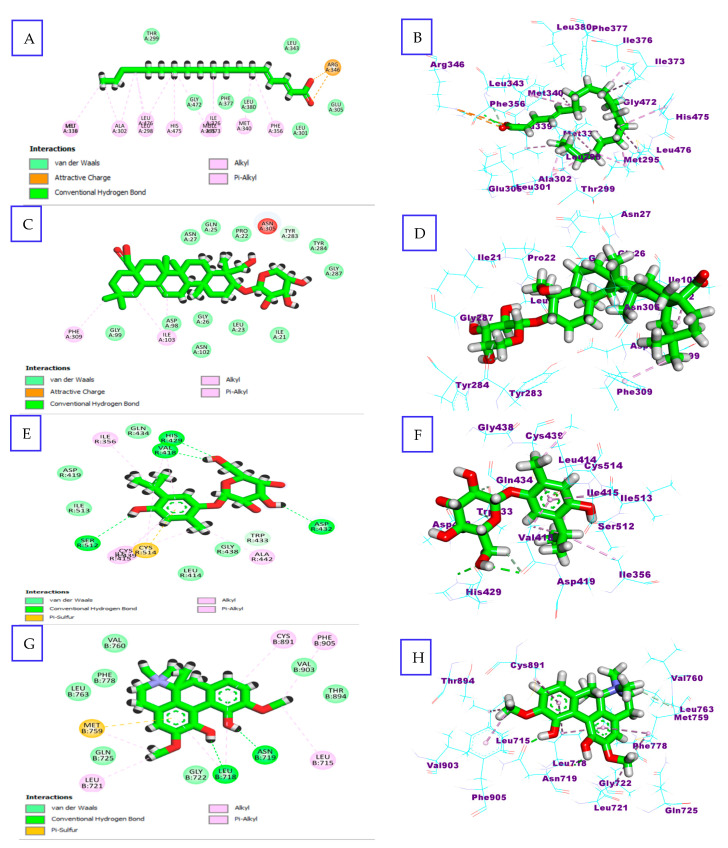
2D and 3D representations of (**A**,**B**) octadecadienoic acid with Estrogen β Receptor (ER β), (**C**,**D**) hederagenin pentoside with Human Gonadotropin-Releasing Hormone Receptor (GnRHR), (**E**,**F**) hymoquinol glucoside with Luteinizing Hormone receptor (LHR), and (**G**,**H**) magnoflorine with Progesterone Receptor (PR).

To further understand the extract activity, MOE software was used to conduct in silico analyses of the LC-MS-annotated compounds (1–20) into the receptors involved in the development of the female genital system. In comparison to the co-crystallized reference for the five proteins, the majority of the compounds displayed remarkable binding affinities (Table 2 and Figure 8). Regarding ERβ, the fatty acids, octadecadienoic acid and palmitic acid, showed the highest affinities, with ΔG = −9.20 and −9.00 kcal/mol, where the two hydrogen bonds were formed between the oxygens of the carboxyl and the crucial amino acid Arg 346. Also, the alkaloids, nigelanoid, nigellidine, and magnoflorine, and thymoquinol derivatives revealed the binding affinities with ΔG ≤ −8.00 kcal/mol, forming hydrogen bonds between their phenolic hydroxyls and the key amino acids, like Leu 339. The same was true with Progesterone Receptors, magnoflorine, E-caffeic acid 3-*O*-glucuronide, and palmitic acid, which exhibited good binding affinities, with ΔG = −8.57, −7.82, and −7.63 kcal/mol, respectively. This study revealed the presence of hydrogen bonds between the compounds and the key amino acids Asn 719, Gln 725, and Arg 766. Also, thymoquinol derivatives, octadecanoic acid, E-caffeic acid 3-*O*-glucuronide, and magnoflorine exhibited noticeable binding affinities to the ligand site of LH, with ΔG = −7.51, −7.31, −6.45, and 6.21 kcal/mol compared to the reference Org 43553 [33]. With the key amino acids of LH receptors, including Val 418, His 429, Gln 434, and Asp 432, the compounds formed several hydrogen bonds. Additionally, the previous compounds as well as hederagenin pentoside showed remarkable binding affinities to the crucial amino acids of GnRH, with ΔG ≤ −7.00 kcal/mol.

## 3. Discussion

This study aimed to investigate the impact of *N. sativa* on the female reproductive system of rats to explore its potential benefits in improving fertility. It is known that there are infinite phytochemical studies on *N. sativa* seeds, but a new study concerned with the effect of *N. sativa* seed ethanol extract on the fertility of immature female rats was needed. Herein, the further HPLC-ESI-MS/MS profiling of *N. sativa* seed ethanol extract was constructed based on the matching of the output LC-MS data with the conventional library database means comparison, including R_t_-values, accurate masses of both molecular ions, and characteristic fragments [25]. The results revealed the identification of thymoquinolglucoside, hederageninpentoside [32], caffeic acid 3-*O*-glucuronide, methoxycinnamic acid, coumaric acid [33], thymoquinolglucuronic acid [32], thymoquinone [31,32], nigelanoid, dihydronigellidine, magnoflorine, hederagenin [28], hederagenin methyl ester [32], tauroside E [32], kaempferol [30], vanillic acid [31], octadecadienoic, octadecenoic, and palmitic acid [25,26]. While several studies have explored the effects of *N. sativa* on males [10,27], there has been limited research on its impact on female reproduction. This study is the first study to focus on the FSH- and LH-like activities of *N. sativa* seed ethanolic extract, as previous studies primarily focused on its estrogenic activity. These findings provide valuable insights into the potential use of *N. sativa* in female reproductive health.

One interesting result of this study is that the *N. sativa* seed extract demonstrated LH-like activity, as evidenced by an increase in LH hormone levels. This finding was also reported by [34,35,36]. The *N. sativa* oils appear to have a direct effect on the hypothalamus, stimulating the secretion of gonadotropin-releasing hormone (GnRH), which in turn stimulates the adenohypophysis (anterior pituitary) to secrete more LH. [34,37]. The long-chain fatty acids (LCFAs), particularly linoleic acid, present in the *N. sativa* seed extract play a crucial role in increasing LH secretion [38]. Previous studies have shown that LCFAs, such as linoleic and oleic acids, enhance the expression of LHβ mRNA in rats while repressing the transcription of FSHβ [39,40]. This explains the increase in the LH serum levels but not the FSH levels. Moreover, the oral administration of *N. sativa* seed extract significantly increases the levels of steroid hormones, including E2 and P4. A possible explanation for the increase in P4 serum levels is the LH-like activity of the *N. sativa* seed extract, which is characterized by an increase in the number of mature CLs, which is the main source of P4 secretion. P4, synthesized from cholesterol [5], plays a vital role in maintaining pregnancy, oocyte release, and implantation [6]. Other hormones, such as prostaglandins, β-adrenergic agents, and prolactin (PRL), are also involved in the regulation of P4 release [41].

Another interesting outcome is that *N. sativa* exhibits estrogenic-like effects, which have the potential to enhance female fertility. The serum Estrogen level was significantly elevated in rats given the *N. sativa* seed extract. Moreover, the estrogenic activity of *N. sativa* seed extract has revealed an increase in the weight of the uterus, the development of variable-sized endometrial folds, an increase in the height of the endometrial epithelium, and dilation of the uterine glands with the infiltration of inflammatory cells. The high Estrogen content of the *N. sativa* extract increases the thickness of the lining epithelium of the uterus, preparing it for the possible implantation of a fertilized egg [8,42,43]. Thymoquinone, a major component of N. sativa seeds, may contribute to this estrogenic effect. A possible explanation for this might be attributed to the high thymoquinone content of *N. sativa* seed extract oils [12,22]. Thymoquinone is one of the main components found in *N. sativa* seed, and it was identified as the most effective compound through LC-HPLC analysis. Thymoquinone plays an integral role in cholesterol synthesis, which is the basic part of steroid synthesis [35]. It was previously reported that the Egyptian *N. sativa* is a part of the thymoquinone chemotype, which is very rich in thymoquinone [44]. Moreover, a previous study revealed that the phenolic compounds and flavonoid content of *N. sativa* have strong estrogenic activity, acting as agonists on human α and β subunits of the Estrogen receptor. This structural similarity to steroid hormones suggests that *N. sativa* could potentially be used as an alternative hormone therapy for post-menopausal conditions by mimicking ovarian endocrine activity. The increase in E2 levels after oral administration of the *N. sativa* seed extract may be attributed to the increase in LH levels, as LH stimulates the growth and development of theca cells, leading to the formation of a dominant follicle, which is a significant source of E2 secretion [45]. These findings indicate that *N. sativa* ethanolic extract may help maintain regular menstrual periods, reproductive cycles, and hormonal balance.

Molecular docking analysis further revealed that *N. sativa* binds with high affinity to Estrogen (ER), Progesterone (PR), and Luteinizing (LHR) hormonal receptors. These receptors play a crucial role in regulating various physiological processes in the body. The docking studies provided valuable insights into the interactions between *N. sativa* compounds and these hormonal receptors. For example, a study examining the potential of *N. sativa* as a natural alternative to synthetic hormone therapy found that thymoquinone, a compound present in *N. sativa,* binds to ER and PR with high affinity. This suggests that thymoquinone may have estrogenic and progestogenic effects [35]. Another study used molecular docking simulations to investigate the binding of thymoquinone to estrogen receptor alpha (ERα). The results demonstrated that thymoquinone binds to ERα with strong affinity, forming hydrogen bond interactions with key amino acid residues [19]. However, the affinity of *N. sativa* compounds for gonadotrophic hormones, such as FSH and LH, is not yet fully understood. This study, being the first to explore the effects of *N. sativa* on female reproduction, investigated the FSH-like and LH-like activities of *N. sativa*. The findings suggest that *N. sativa* compounds may have potential as natural alternatives or supplements for hormone therapy, especially in the treatment of reproductive and gynecological disorders. This is particularly important considering the associated risks of synthetic hormone replacement therapy. However, further research is needed to fully comprehend the mechanisms of action of *N. sativa* compounds and their impact on the regulation of hormonal receptor genes and protein expressions. It is through continued investigation that we can gain a deeper understanding of the potential benefits of *N. sativa* in hormone-related therapies.

## 4. Materials and Methods

### 4.1. Plant Material

*N. sativa* seeds were collected from the cultivated plants section of medicinal plants in the Orman Garden, Egypt (30°01′45″ N 31°12′47″ E) in the early morning of April 2021. Senior botanist Mohamed A. Gibali of the El-Orman Botanic Garden in Giza, Egypt, performed the seed authentication. The El-Orman Botanic Garden Herbarium received the seed sample (Y100812-NS-S086). For the seed preparation, the seeds were cleaned under running tap water for 10 minutes, rinsed twice with distilled water, and air-dried in an oven at 40 °C overnight.

### 4.2. Extraction Processes

The *N. sativa* seeds (500 g) were ground using an electrical grinder. The ground *N. sativa* powder was macerated with a sufficient volume of 100% absolute ethanol (2 L) for 7 days. This process was repeated 3–5 times until the solution was nearly clear. The macerated plant with ethanol was filtered using a Buchner funnel lined with gauze between small pieces of cotton; the beaker was attached with a vacuum pump to facilitate the filtration process; the filtrate of *N. sativa* was dried using a rotary evaporator at 40 °C (Heidolph, Schwabach, Germany). The evaporated extract (75 g) was collected and kept in the refrigerator until it was used for phytochemical analysis and biological assays [46].

### 4.3. HPLC-ESI-MS/MS Analysis

With an ExionLC AC device for separation and a SCIEX Triple Quad 5500+ MS/MS system (Framingham, MA, USA) with electrospray ionization (ESI) for detection, the extract was analyzed employing the LC-ESI-MS/MS. A 4.6 × 150 mm, 3 µm Ascentis^®^ C18 column (Darmstadt, Germany) was used for the separation. Two eluents were used in the mobile phases (B, LC-grade acetonitrile and A, 0.1% formic acid). The following was the schedule for the mobile phase gradient: 10% B, 10–90% B, 90% B, and 10% A at 0–2, 2–30, 30–36, and 36.1–40 mins, respectively. With an injection volume of 10 µL, the flow rate was 0.7 mL/min. To perform an MS/MS analysis, the negative ionization technique was utilized with scan rates ranging from 50 to 1000 *m*/*z* for MS2, with the following settings: declustering potential: −80; collision energy: −35; collision energy spread; curtain gas: 25 psi; ion spray voltage: −4500 V; source temperature: 500 °C. Ion source gas 1 and 2 were 45 psi. The MS-DIAL program version 4.70 was utilized to identify the components [29].

### 4.4. Biological Assays

#### 4.4.1. Chemicals, Drugs, and Kits

The analytical-grade ethanol used for plant extraction was purchased from El-Gomhouria Co. in Cairo, Egypt. Pregnant mare’s serum gonadotropin (PMSG, Folligon^®^) with a concentration of 1000 IU/vial was obtained from Intervet International B.V. in Boxmeer, NOORD-BRABANT, Netherlands. Human chorionic gonadotropin (hCG, Epifasi^®^) in lyophilized ampoules containing 5000 IU of hCG was purchased from Egyptian Int. Pharmaceutical Industries Co. in Cairo, Egypt. Estradiol benzoate (Folone^®^) in an oily solution with a concentration of 5 mg/mL/ampoule was purchased from Misr Company for Pharmaceuticals in Cairo, Egypt. Thiopental Sodium^®^ with a strength of 500 mg was purchased from EIPICO in Cairo, Egypt. Tween 80 and 10% formalin were purchased from Sigma Aldrich in St. Louis, MO, USA. Serum levels of follicle-stimulating hormone, estradiol, and Progesterone were determined using ELISA kits (IMMUNOSPEC, Bioquote Ltd., York, UK and BIOS, Microwell Diagnostic Systems, Chemux Bioscience Inc., California, USA).

#### 4.4.2. Experimental Animals

The protocol of this study was approved by the Medical Research Ethics Committee and Animal Care and Use Committee (ACUC), National Research Centre, Egypt, with 16/233 reference numbers for notice of approval. Immature female albino rats (Ratus ratus), weighing 70–80 g (age less than 21 days), were obtained from the Animal House colony, National Research Centre. Animals were housed in stainless steel, wire-meshed plastic cages under standard conditions of humidity, temperature (25 ± 2 °C), and light/dark cycle (12 h/12 h). The rats were fed a formulated ration in the form of pellets. The experimental rats drank clean tap water offered ad libitum. 

#### 4.4.3. Preliminary Study 

In the preliminary study, the extract did not show any signs of toxicity when administered to rats at a dose of (2000 mg/kg b.w.). Three different doses, 1/5, 1/10, and 1/20 of the high dose used in rats (2000 mg/kg b.w.), were tested at doses of 100, 200, and 400 mg/kg b.w., along with a reference drug (PMSG). All doses resulted in histological changes, such as an increase in the number of mature follicles and thickening of the endometrium, indicating a positive impact on fertility (Figure S13). The higher doses (200 and 400 mg/kg body weight) were more effective than the lower dose (100 mg/kg body weight). Among the higher doses, both 200 mg/kg and 400 mg/kg body weight showed similar results. Therefore, the smaller dose of 200 mg/kg b.w. was selected for further studies.

*N. sativa* ethanolic extract was assessed for its hormonal-like effect on the reproductive tract via three distinct studies, as follows: 1. FSH-like activity; 2. LH-like activity; and 3. estrogenic activity.

#### 4.4.4. FSH-like Activity

The FSH-like activity of *N. sativa* extract was determined according to the method described by [47], which measures ovarian hypertrophy resulting from exogenous FSH administration in immature female rats. In brief, twenty-one female immature rats were divided into three groups (*n* = 7) and treated as follows: the control group received 0.5 mL of normal saline orally. The positive control group was injected with a single dose of PMSG intraperitoneally (20 IU/0.5 mL) [47]. The last group received an ethanolic extract of *N. sativa* orally (200 mg/100 g b.wt.) [48]. The animals were treated for 4 days, blood samples were collected, and then the rats were decapitated on the 5th day to be used for morphological and histological assessment. 

#### 4.4.5. LH-like Activity

The LH-like activity of *N. sativa* was assessed according to the method described by [49]. The control group (Group 1) received 0.5 mL of normal saline for 4 days after a single intraperitoneal injection of 20 IU/0.5 mL of PMSG (Folligon^®^). The positive control group (Group 2) received 20 IU/0.5 mL of hCG for 4 days after the same injection of PMSG. The third group (Group 3) was treated with an ethanolic extract of *N. sativa* orally (200 mg/100 g b.wt.) for 4 days after i.p. injection of a single dose of PMSG (20 IU/0.5 mL). After blood collection, 48 h after the last dose administration, all animals were decapitated and kept for morphological and histological assessment of the reproductive organs.

#### 4.4.6. Estrogenic Activity (Ovariectomized Rats)

The estrogenic effect of *N. sativa* was analyzed according to [50]. A total of 21 immature, female, albino rats were ovariectomized after 24 h of fasting under anesthesia using an i.p. injection of thiopental sodium (50 mg/kg b.wt.). After recovery about 10 days after the surgical operation, the animals were classified into 3 groups (7 rats/group). Group 1 (control) was injected with 0.5 mL of normal saline. Group 2 (positive control) rats were injected i.p. with a single dose of estradiol benzoate (Folone^®^, 5 mg/mL/ampoule in oily solution). Group 3 rats were treated with ethanolic extract of *N. sativa* extract (200 mg/100 g b.wt.). After one week from the beginning of the treatment, adequate amounts of blood were collected, and then the animals were decapitated and the uteri were excised from the surrounding tissues. Each uterus was separated and weighed. The relative weights were expressed as g per 100 g body weight.

#### 4.4.7. Blood Sample Collection and Morphological and Metric Assessment of the Reproductive Tract

The blood was collected from the inner corner of the eye into tubes containing heparin as an anticoagulant. The collected blood was then centrifuged at 3000 rpm for 10 min, separating the plasma, which contains the hormones of interest. The plasma samples were stored at −20 °C until they were ready for analysis. After decapitation, the rats’ bodies were opened along the mid-line, and their genitalia were carefully removed and freed from the surrounding fat. The genitalia were photographed using a digital camera and weighed using a precise balance. To determine the levels of specific hormones, the following ELISA kits were used:

- The Rat FSH ELISA Kit from Wuhan Fine Biotech in Wuhan, Hubei, China was used to measure FSH levels.
- LH levels were measured using an LH ELISA kit (Ref. no. E29-118, Lot. Number: 117041404) from IMMUNOSPEC in Seattle, Washington, DC, USA.
- An Estradiol (E2) ELISA kit (Catalog No. 10009, Lot. Number: 118031903) from BIOS, Microwell Diagnostic Systems Chemux Bioscience, Inc. in the Netherlands was used to measure estradiol levels.
- Progesterone levels were determined using an ELISA kit (Ref. no. 10005, Lot. Number: 118042304) from Chemux BioScience, INC in South San Francisco, CA, USA.

All of the procedures followed the instructions provided in the respective manufacturers’ manuals.

#### 4.4.8. Histological Examination

At the end of each experiment, tissue specimens from the ovaries and uteri of control and treated rats were fixed in 10% formalin overnight, washed and dehydrated in ascending concentrations of alcohols, and then cleared in xylene and embedded in paraffin. The paraffin blocks were sectioned at 3–5 µm thickness using a microtome and stained through H&E [51]. The histological changes were determined under an Olympus CX41 light microscope (Tokyo, Japan).

### 4.5. Molecular Docking Studies

Molecular Operating Environment (MOE) software version 2015.10 was used for docking research. From the protein data bank, the catalytic domains of Human Gonadotropin-Releasing Hormone Receptor (GnRH1R) (PDB ID: 7BR3) [52], Human Luteinizing Hormone Receptor (LHR) (PDB ID: 1XWD) [33], Estrogen Receptor β (ERβ) (PDB ID: 1X7B) [53], and Human Progesterone Receptor (PDB ID: 1E3K) [54] were extracted. The PDB bank was used to choose the conformation as it contains a vast collection of experimentally determined protein structures, including this study’s target proteins. Additionally, the quality and reliability of the protein structures available in the PDB bank are important considerations. Structures with higher resolution and accurate experimental techniques are preferred to minimize potential errors or inaccuracies in the docking process. The crystal structures were processed by removing undesirable solvents, ligands, and cofactors, employing the default MOE “QuickPrep” module settings, and then applying the “Site Finder” feature. The possible binding pockets encompassing the crucial residues were prepared. The co-crystallized ligands were re-docked in the identified binding site for docking process validation, showing the majority of the critical interactions at an acceptable RMSD. The database file (.mdb) of the energy-minimized identified compounds using HPLC-MS-MS (1–20) was built and docked in the binding site. The results were presented as S-scores with RMSD values ≤ 2 Å (Table 2), and the 2D and 3D representations of the best conformers with the most suitable protein were inspected.

### 4.6. Statistical Analysis

All data were obtained as the mean ± SEM and compared through one-way analysis of variance (ANOVA) followed by Tukey’s multiple comparison test to compare the significance between the different groups; *p* < 0.05 was considered significant. The Graph Pad Prism 9 software package (GraphPad Software, Inc., San Diego, CA, USA) was used for performing all of the calculations.

## 5. Conclusions

This study highlights the potential of *N. sativa* seed extract as a natural remedy for managing female reproductive problems and enhancing fertility. The findings suggest a strong interaction between the extract’s components and hormonal receptors, indicating its potential effectiveness. Tauroside E, nigellidine, and thymoquinol glucoside, as well as other compounds, were identified via HPLC-ESI-MS/MS analysis. The MOE assessment showed that *N. sativa* extract components, particularly these chemicals, strongly interacted with ER and LHR target proteins. However, further research is required to explore the underlying mechanisms and expand the scope of the investigation to encompass other areas of reproductive health. Further studies are needed regarding the other forms of the plant and different extraction methods. Additionally, further studies employing a variety of methodologies, including genetic and proteomic analyses, would be beneficial for gaining a more comprehensive understanding of the effects of *N. sativa* seed extract on hormonal receptors and their relationship with the regulation of hormonal receptors’ gene and protein expressions. 

## Figures and Tables

**Table 1:** The metabolites tentatively identified from *N. sativa* ethanol extract observed using HPLC–MS/MS, with their retention time.

No	Rt (min)	Tentatively Identified Compounds	Classes	[M-H]^−^ *m*/*z*	MS^2^ Major Production	Refs.
1	3.615	Kaempferol	Flavanol	285.04		[30]
2	8.040	Vanillic acid	Phenolic acid	167.02		[31]
3	8.180	Magnoflorine	Alkaloid	340.08		[28]
4	8.423	Thymoquinol glucoside	Monoterpene glycoside	325.05	310.07, 282.09, 163.00	[32]
5	10.230	Nigellidine	Alkaloid	295.17	277.28, 171.17	[28]
6	13.592	Hederagenin aglycone	Saponin	471.73	393.02	[32]
7	14.090	Hederagenin pentoside	Saponin	603.31		[32]
8	16.437	Tauroside E	Saponin	749.20	603.29, 471.36	[32]
9	18.390	Hederagenin methyl ester	Saponin	485.21	467.56, 439.83	[32]
10	27.012	Dihydronigellidine	Alkaloid	293.07	265.05, 237.05, 222.09	[28]
11	30.300	Methoxycinnamic acid	Phenolic acid derivative	177.87	159.90	[31]
12	31.689	Thymoquinone	Monoterpene	163.98	117.92	[31,32]
13	32.932	Caffeic acid 3-*O*-glucuronide	Phenolic acid derivative	355.3	179.14, 163.15	[31]
14	33.473	(*E*)-*p*-Coumaric acid	Phenolic acid	163.07	119.00	[31]
15	33.574	Malic acid	Fatty acid	132.84	115.88	[25]
16	33.622	Thymoquinol glucuronic acid	Monoterpene glycoside	339.16	163.12	[32]
17	34.931	Octadecadienoic acid	Fatty acid	279.24		[26]
18	34.289	Nigelanoid	Alkaloid	312.11	293.86, 275.86, 183.04, 119.08	[31]
19	37.430	4-Vinylphenol	Phenol	118.90	101.89	[30]
20	38.654	Octadecenoic acid	Fatty acid	281.22		[25]
21	38.489	Palmitic acid	Fatty acid	255.29		[26]

**Table 2:** Results of docking simulations of the identified compounds of the leaf extract to the active sites of the viral proteins.

Compound	ΔG a (kcal/mol)				
	E2	FSH	LH	GnRH	P4
4-Vinyl phenol	−4.89	−3.31	−4.37	−4.32	−4.66
(*E*)-*p*-coumaric acid	−5.60	−3.35	−4.92	−4.62	−4.84
*E* -Caffeic acid 3-*O*-glucuronide	−7.75	−4.85	−6.45	−7.20	−7.82
Hederagenin methyl ester	----	----	----	----	----
Hederagenin pentoside	----	----	----	−7.53	----
Hederagenin aglycone	----	----	----	----	----
Kaempferol	−7.34	−4.15	−6.09	−6.13	−6.57
Magnoflorine	−8.15	−4.45	−6.26	−7.00	−8.57
Malic acid	−4.93	−3.84	−4.58	−4.21	−4.44
Methoxycinnamic acid	−5.71	−3.78	−5.08	−4.78	−5.12
Nigelanoid	−8.80	−4.36	−6.04	−6.00	−6.92
Nigellidine	−8.16	−4.34	−6.02	−6.31	−6.74
Octadecadienoic acid	−9.20	−4.74	−6.66	−7.49	−7.17
Octadecanoic acid	−8.56	−5.11	−7.31	−7.28	−6.57
Palmitic acid	−9.00	−4.88	−6.75	−6.52	−7.63
Tauroside E	----	----	----	----	----
Thymoquinol glucoside	−8.10	−4.27	−7.51	−7.16	−7.54
Thymoquinol glucuronic acid	−8.23	−4.27	−6.95	−6.98	−7.53
Thymoquinone	−6.05	−3.54	−5.40	−4.92	−5.27
Vanillic acid	−5.72	−3.21	−4.89	−4.65	−5.06
E2 ligand	−7.89	----	----	----	----
FSH ligand	----	−6.21	----	----	----
Org 43553	----	----	−8.59	----	----
GnRH ligand	----	----	----	−9.92	----
P4 ligand	----	----	----	----	−7.64

## Data Availability

Data are contained within the article.

## Supplementary Materials

The following supporting information can be downloaded at: https://www.mdpi.com/article/10.3390/molecules29030735/s1, Figure S1: Ms/Ms spectra of magnoflorine; Figure S2: Ms/Ms spectra of nigellidine; Figure S3: Ms/Ms spectra of dihydronigellidine; Figure S4: Ms/Ms spectra of nigelanoid; Figure S5: Ms/Ms spectra of tauroside E; Figure S6: Ms/Ms spectra of hederagenin methyl ester; Figure S7: Ms/Ms spectra of thymoquinolglucoside; Figure S8: Ms/Ms spectra of thymoquinone; Figure S9: Ms/Ms spectra of thymoquinolglucuronic acid; Figure S10: Ms/Ms spectra of caffeic acid 3-*O*-glucuronide; Figure S11: Ms/Ms spectra of octadecadienoic acid; Figure S12: Ms/Ms spectra of octadecenoic acid; Figure S13: Female immature rats.
