# Supplementary material for: Enhancement of Female Rat Fertility via Ethanolic Extract from Nigella sativa L. (Black Cumin) Seeds Assessed via HPLC-ESI-MS/MS and Molecular Docking
Source: Molecules. 2024 Feb 5;29(3):735. doi: 10.3390/molecules29030735 (PMC10856701; doi:10.3390/molecules29030735)
Supplement: Supplementary file 1 [file molecules-29-00735-s001.zip › molecules-2799427-supplementary.pdf]

Supporting data

Enhancement of Female Rat Fertility via Ethanolic Extract from *Nigella sativa* L. (Black Cumin) Seeds Assessed via HPLC-ESI-MS/MS and Molecular Docking

Ahmed M. Nagy[1*], Abdel-Hameed M. Fayed[2], Asmaa S. Abd Elkarim[3], Tushar C. Sarker[4], Ahmed M. Abd-ElGawad[5], Abdelsamed I. Elshamy[6], Abdelmohsen M. Hammam[1.]

1 Department of Animal Reproduction & AI, Veterinary Research Institute, National Research Center, Cairo 12622, Egypt; am.hammam@nrc.sci.eg
2 Department of Pharmacology, National Research Center, Cairo 12622, Egypt; fayed.nrc@gmail.com
3 Chemistry of Tanning Materials and Leather Technology Department, National Research Center, Cairo 12622, Egypt; as.abdel-maksoud@nrc.sci.eg
4 Texas A&M AgriLife Research Center, Overton, TX 75684, USA; tushar.sarker@ag.tamu.edu
5 Plant Production Department, College of Food & Agriculture Sciences, King Saud University, P.O. Box 2460, Riyadh 11451, Saudi Arabia; aibrahim2@ksu.edu.sa
6 Department of Natural Compounds Chemistry, National Research Center, Cairo 12622, Egypt
* Correspondence: an.nagy@nrc.sci.eg (A.M.N.); ai.el-shamy@nrc.sci.eg or elshamynrc@yahoo.com (A.I.E.)

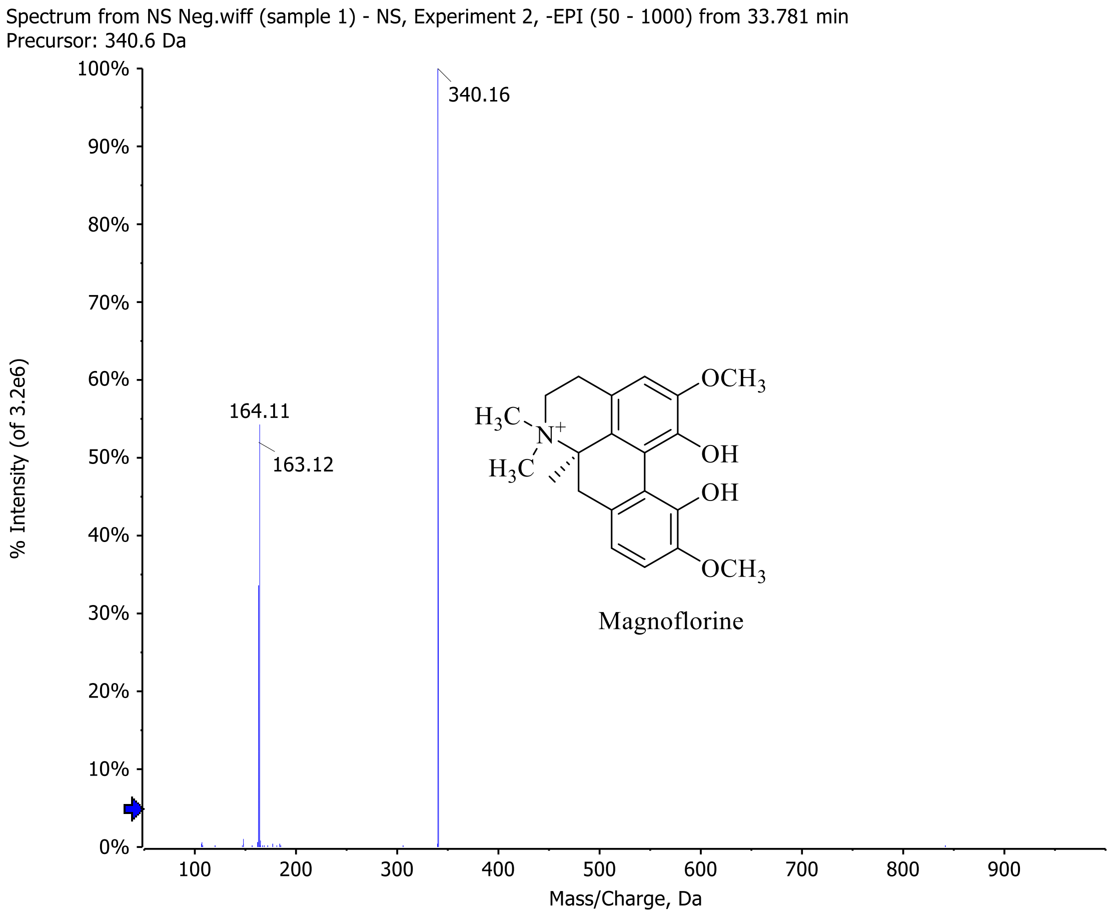

Figure S1: Ms/Ms spectra of Magnoflorine

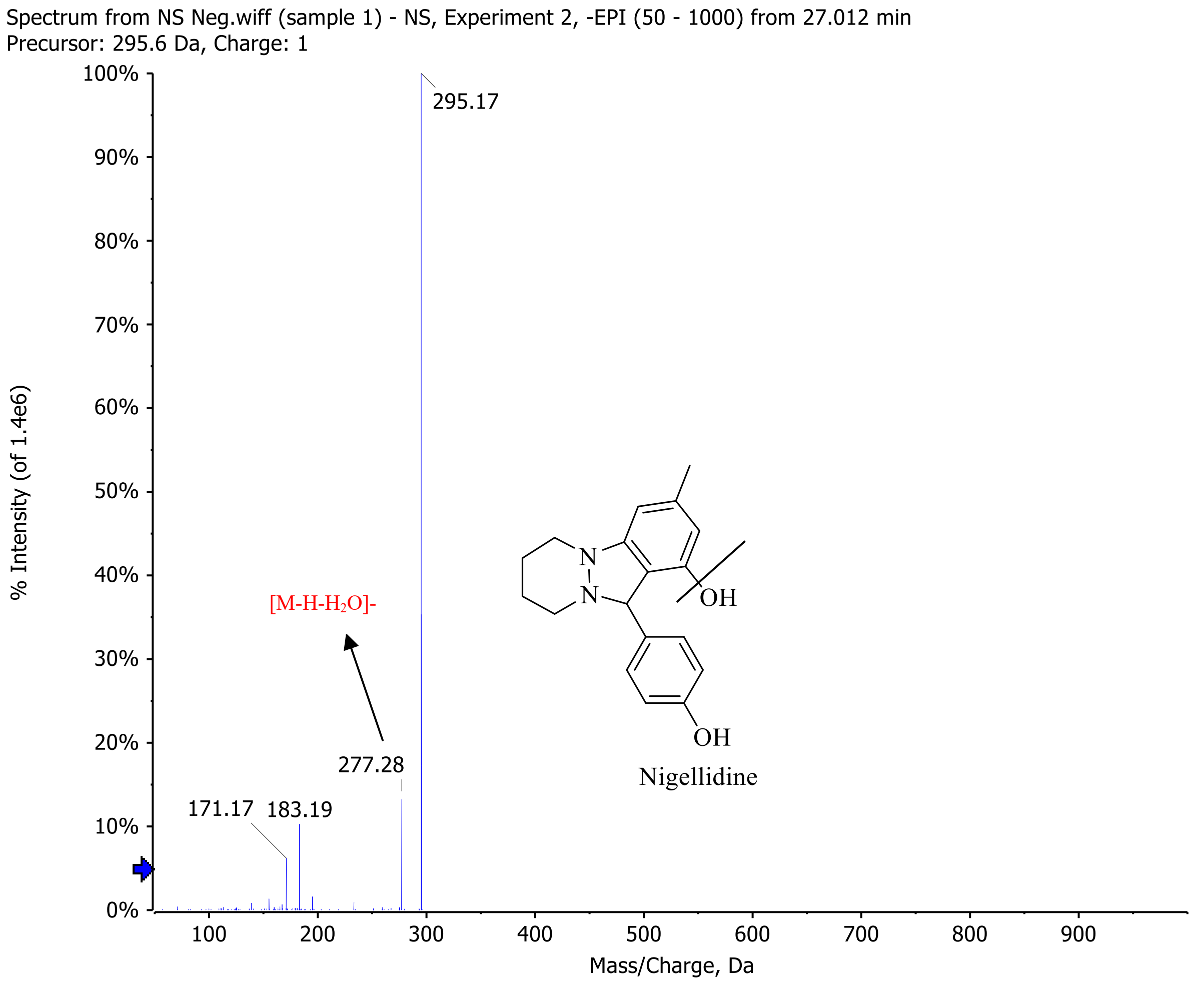

Figure S2: Ms/Ms spectra of Nigellidine

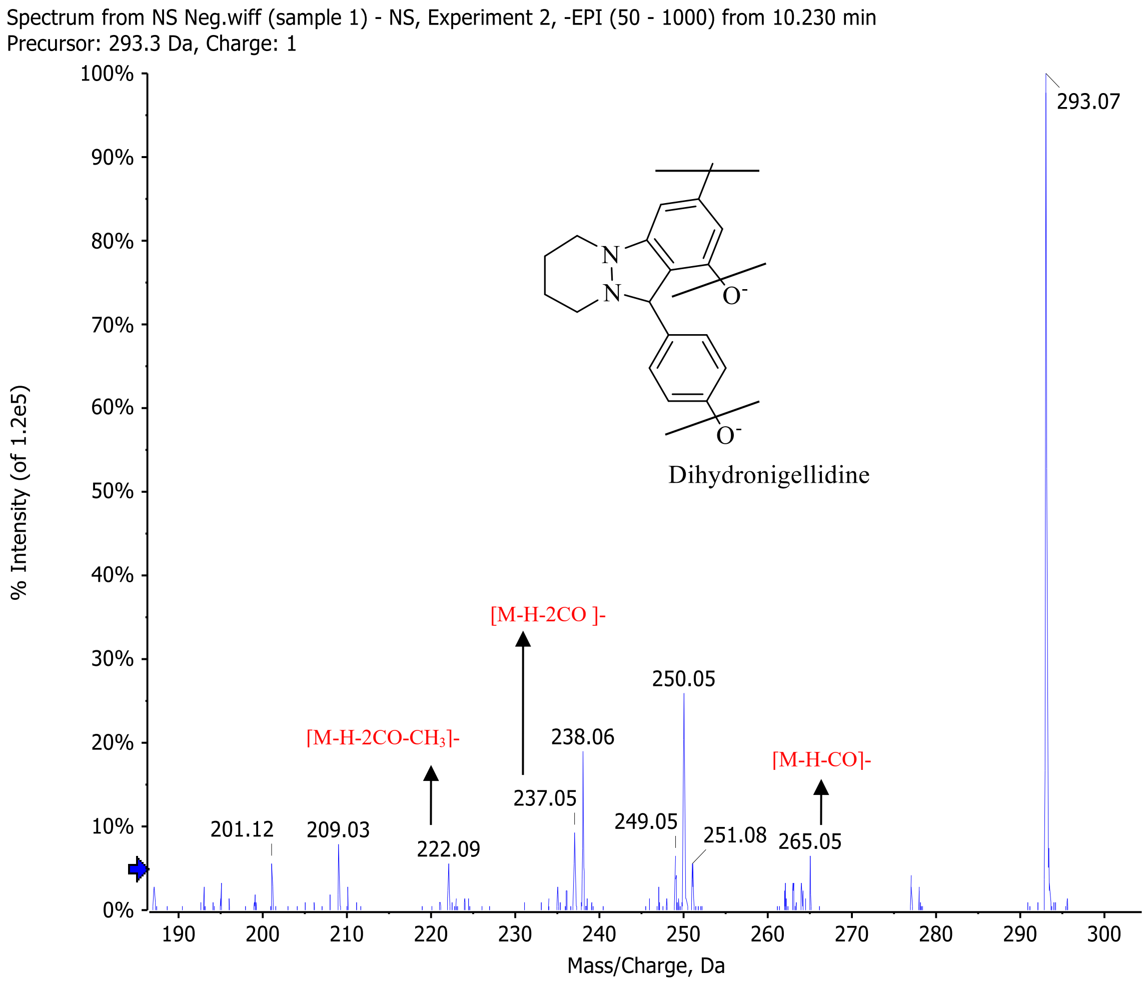

Figure S3: Ms/Ms spectra of Dihydronigellidine

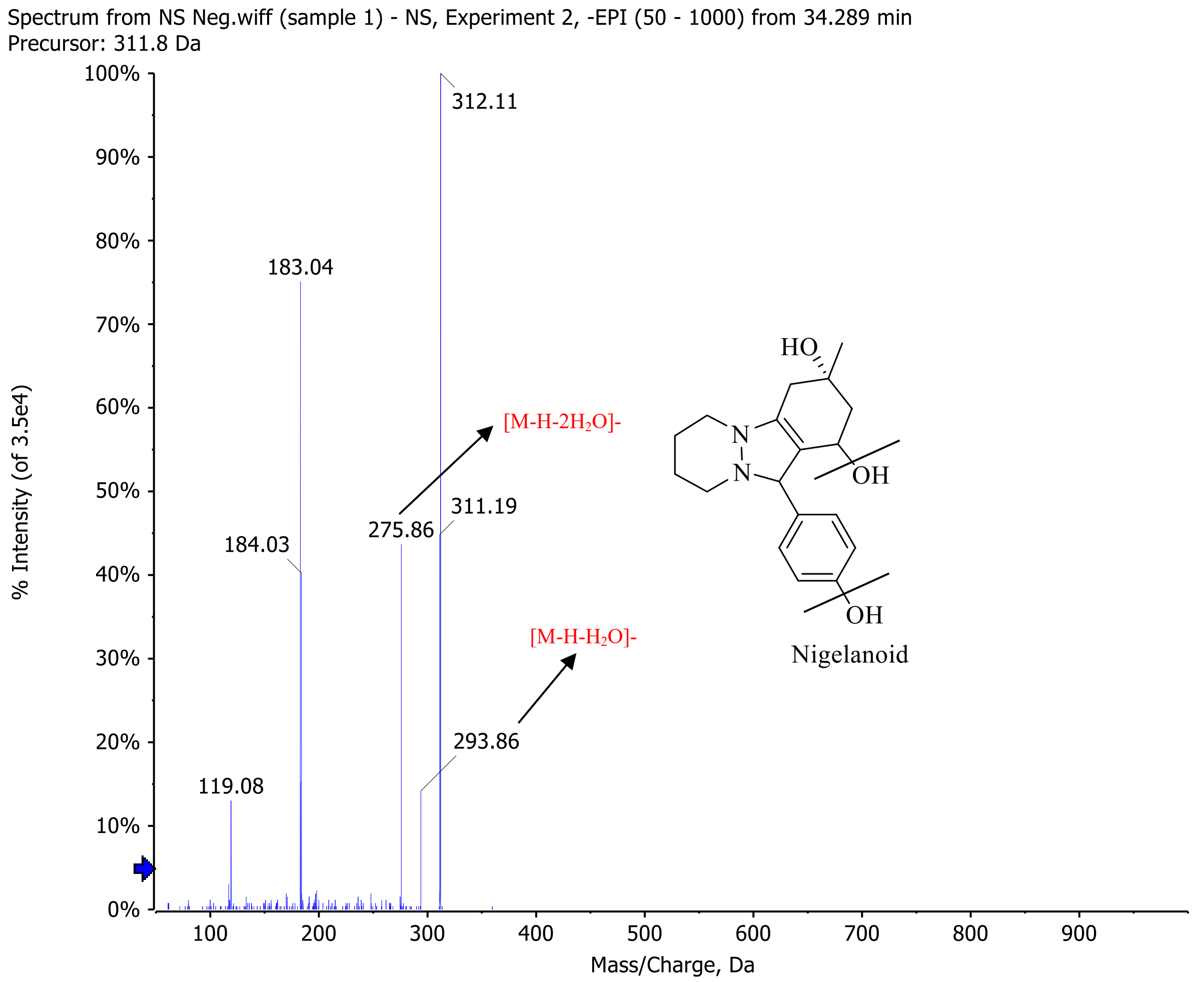

Figure S4: Ms/Ms spectra of Nigelanoid

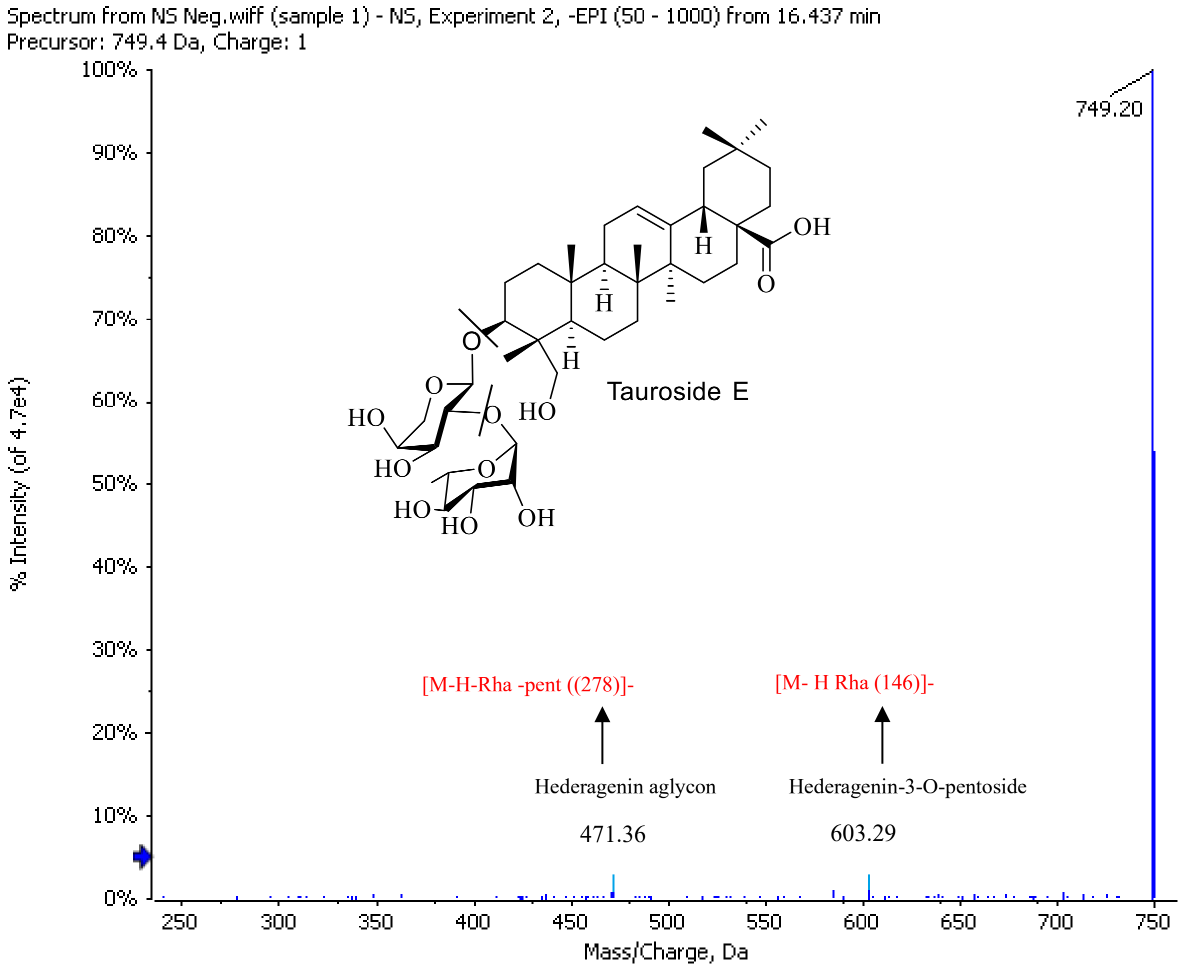

Figure S5: Ms/Ms spectra of Tauroside E

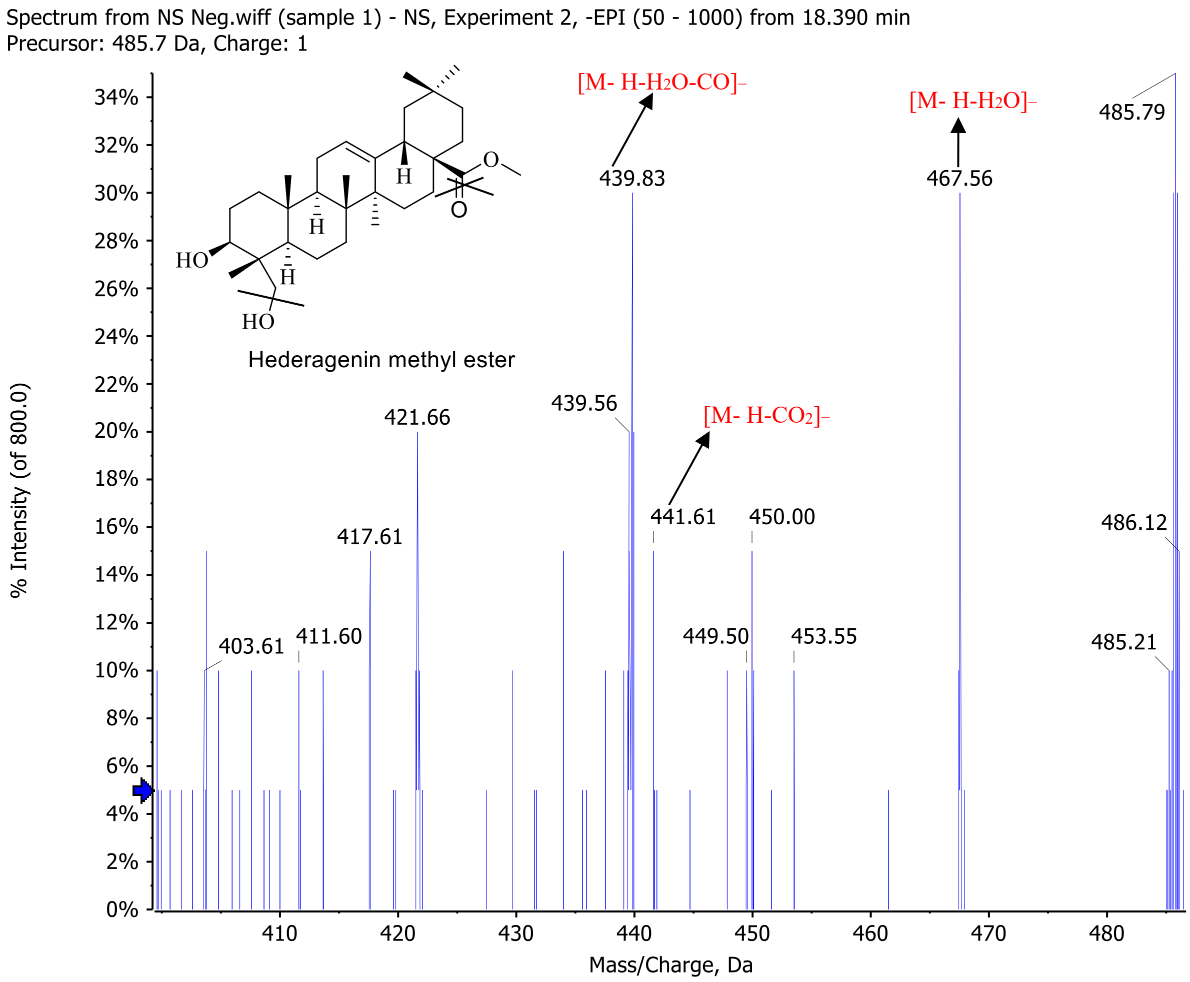

Figure S6: Ms/Ms spectra of Hederagenin methyl ester

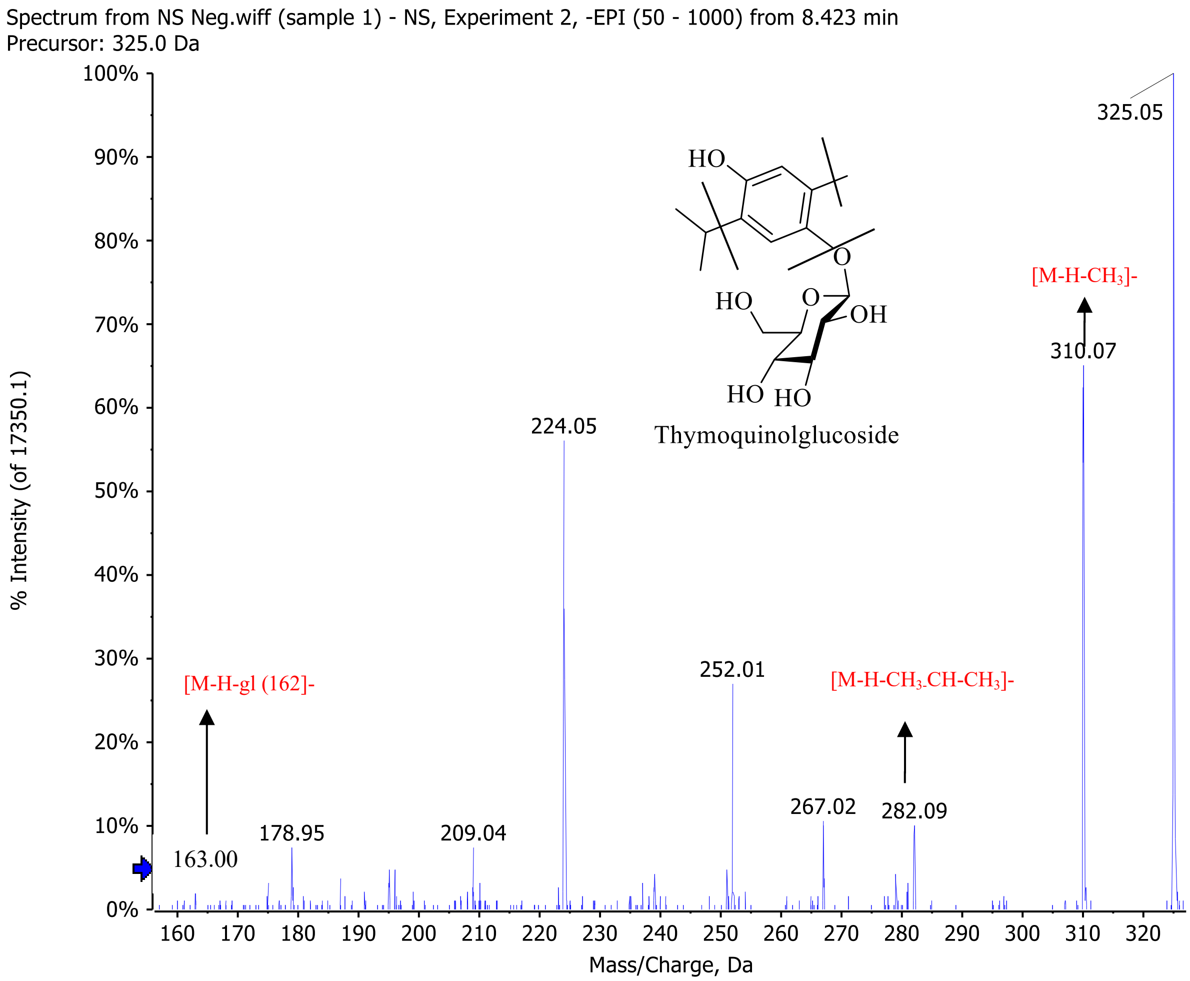

Figure S7: Ms/Ms spectra of Thymoquinolglucoside

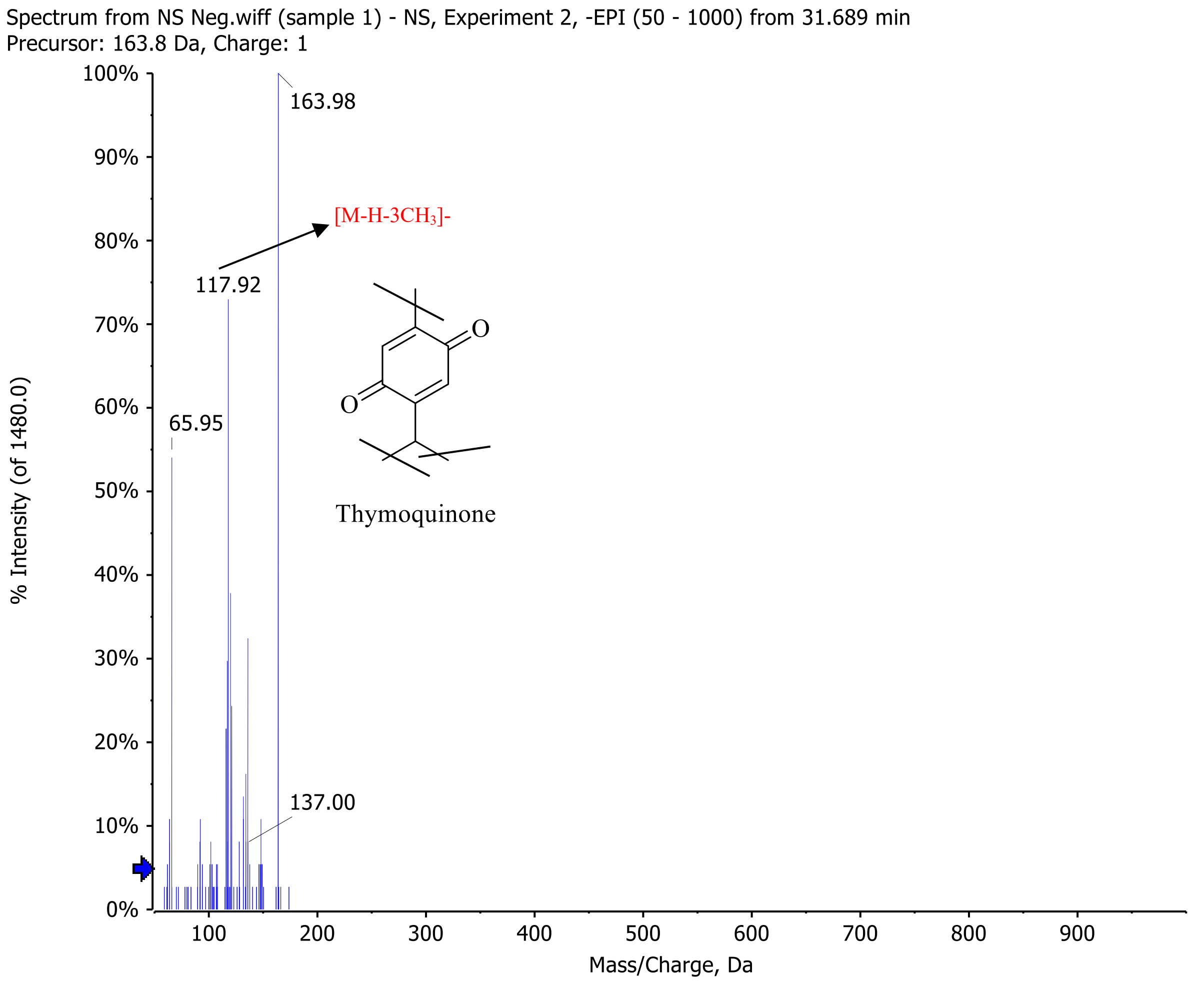

Figure S8: Ms/Ms spectra of Thymoquinone

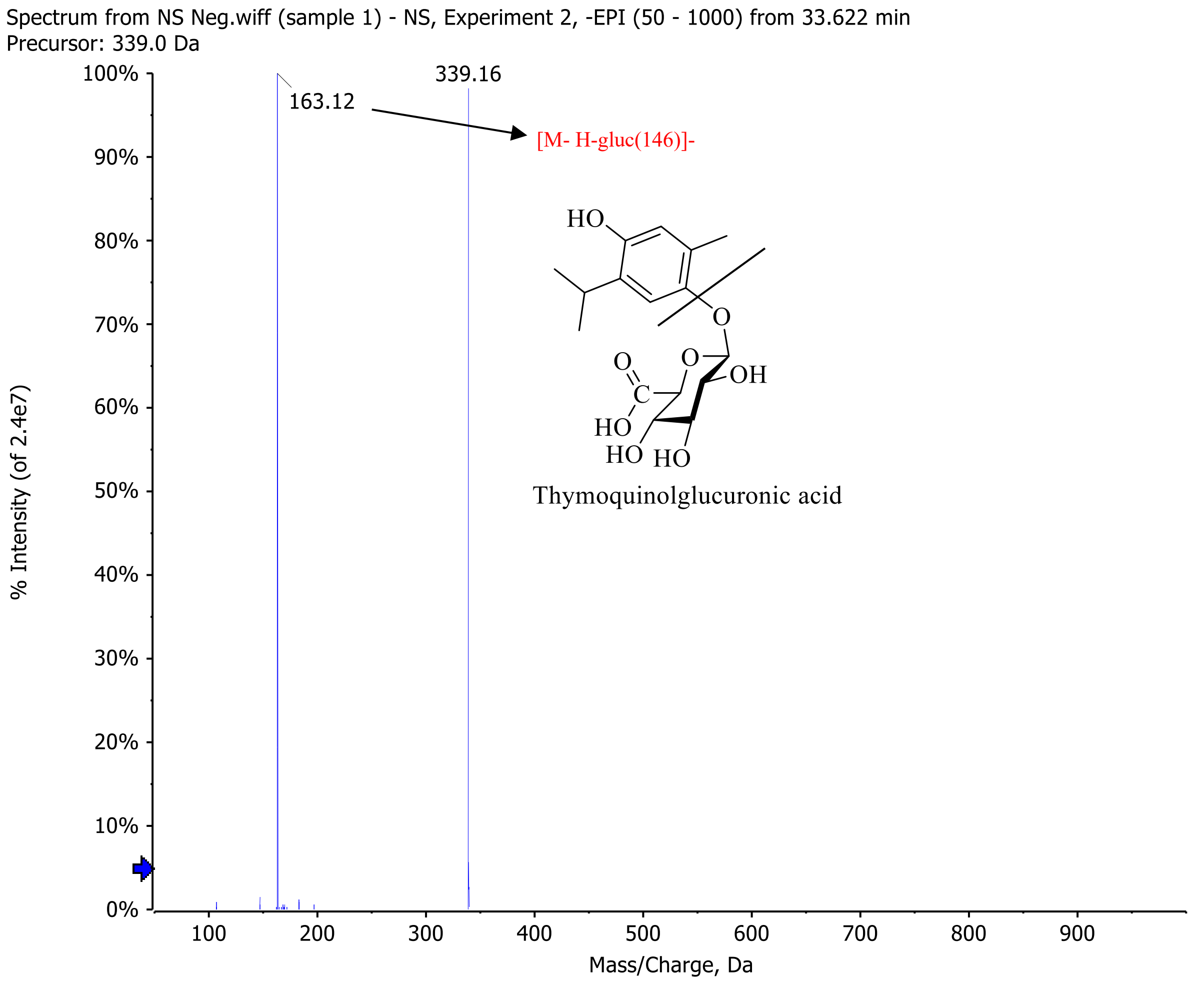

Figure S9: Ms/Ms spectra of Thymoquinolglucuronic acid

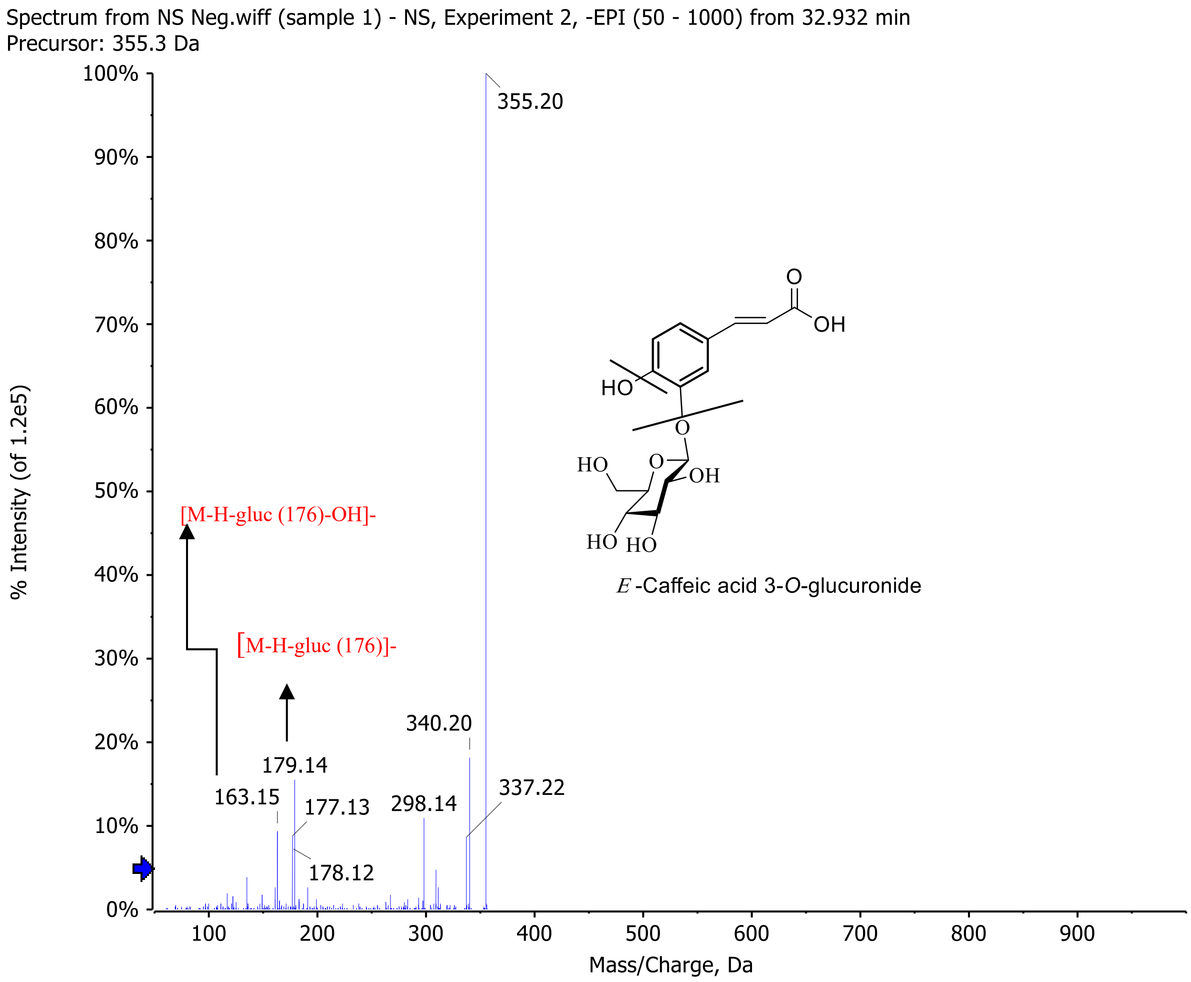

Figure S10: Ms/Ms spectra of Caffeic acid 3-*O*-glucuronide

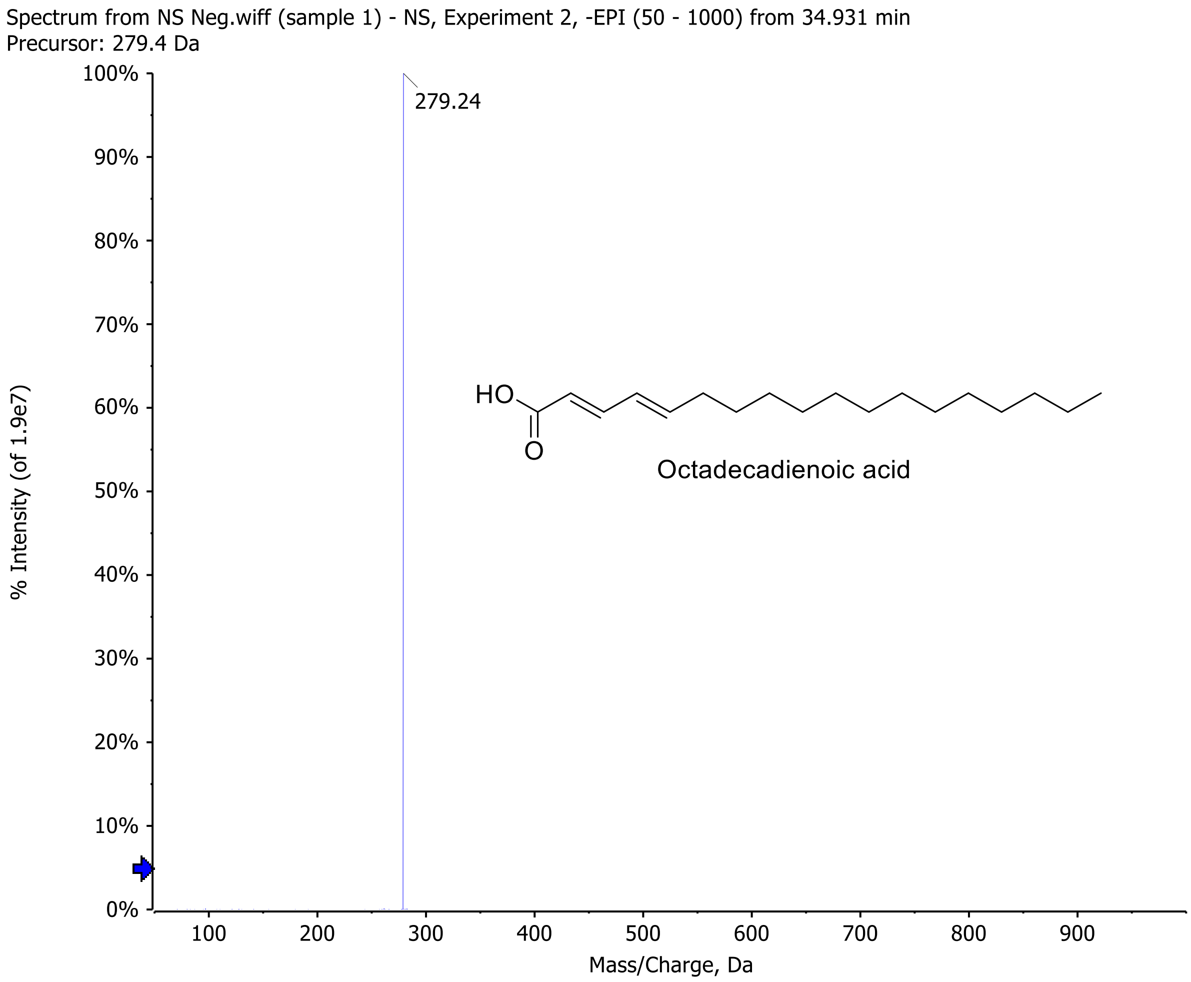

Figure S11: Ms/Ms spectra of Octadecadienoic acid

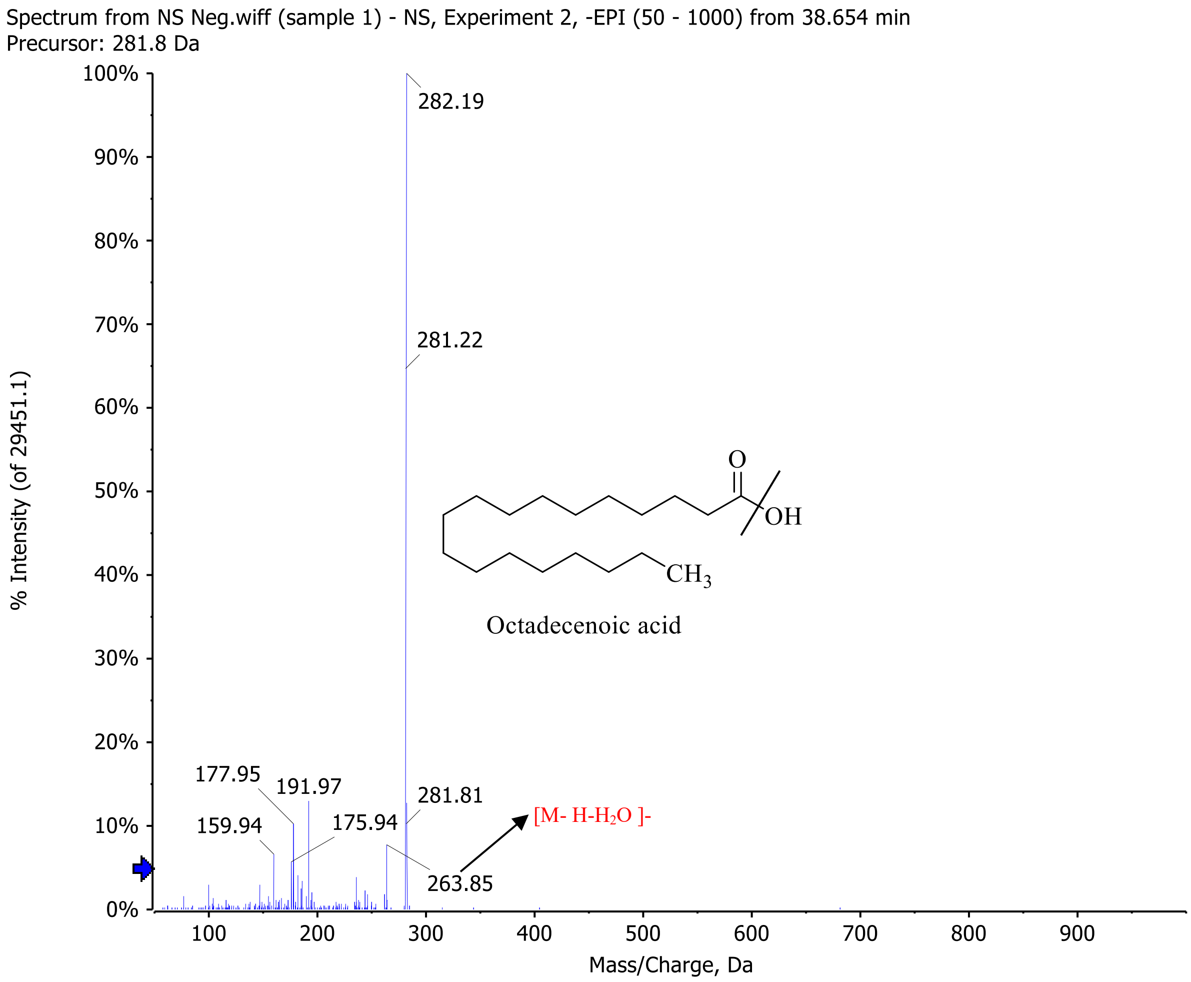

Figure S12: Ms/Ms spectra of Octadecenoic acid

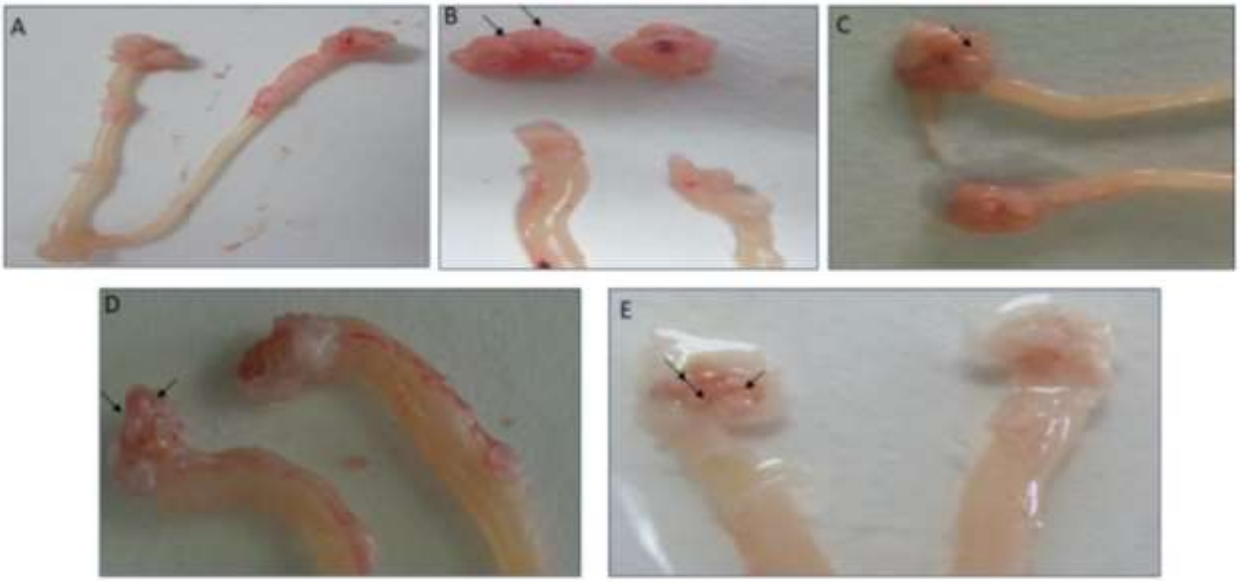

Figure S13: Female immature rats (**A**) Control-ve group treated with saline, the uterus is elongated and thin, and the ovaries are small and contain small numbers of follicles. (**B**) Control FSH+ve group treated with 20 IU PMSG. The uterine body and horns are wide in diameter and filled with large quantities of fluids, the ovaries are large and contain large numbers of large follicles. (**C**) *N. sativa* -treated group with dose 100mg /kg b.w. the uterus is thin, and the ovaries contain small numbers of follicles. (**D**-**E**) *N. sativa* -treated groups with doses (200 and 400 mg /kg b.w.) the uterine body large in diameter, filled with uterine secretions and the ovaries contained large number of follicles.
